# Phosphorylation of enteroviral 2A^pro^ at Ser/Thr125 benefits its proteolytic activity and viral pathogenesis

**DOI:** 10.1002/jmv.28400

**Published:** 2022-12-22

**Authors:** Yuya Wang, Wenjia Zou, Yan Niu, Sanyuan Wang, Bangtao Chen, Rui Xiong, Peng Zhang, Zhijun Luo, Yong Wu, Changfa Fan, Zhaohua Zhong, Ping Xu, Yihong Peng

**Affiliations:** ^1^ Department of Microbiology, School of Basic Medical Sciences Peking University Health Science Center Beijing China; ^2^ Department of Medicinal Chemistry, School of Pharmaceutical Sciences Peking University Health Science Center Beijing China; ^3^ Jiangxi Provincial Key Laboratory of Tumor Pathogens and Molecular Pathology, Queen Mary School Nanchang University Jiangxi Medical College Nanchang China; ^4^ Division of Animal Model Research, Institute for Laboratory Animal Resources National Institutes for Food and Drug Control Beijing China; ^5^ Department of Microbiology Harbin Medical University Harbin China

**Keywords:** eIF4GI, enteroviral 2A^pro^, phosphorylation, proteolytic activity, viral pathogenesis

## Abstract

Enteroviral 2A proteinase (2A^pro^), a well‐established and important viral functional protein, plays a key role in shutting down cellular cap‐dependent translation, mainly via its proteolytic activity, and creating optimal conditions for *Enterovirus* survival. Accumulated data show that viruses take advantage of various signaling cascades for their life cycle; studies performed by us and others have demonstrated that the extracellular signal‐regulated kinase (ERK) pathway is essential for enterovirus A71 (EV‐A71) and other viruses replication. We recently showed that ERK1/2 is required for the proteolytic activity of viral 2A^pro^; however, the mechanism underlying the regulation of 2A^pro^ remains unknown. Here, we demonstrated that the 125th residue Ser125 of EV‐A71 2A^pro^ or Thr125 of coxsackievirus B3 2A^pro^, which is highly conserved in the *Enterovirus*, was phosphorylated by ERK1/2. Importantly, 2A^pro^ with phosphor‐Ser/Thr125 had much stronger proteolytic activity toward eukaryotic initiation factor 4GI and rendered the virus more efficient for multiplication and pathogenesis in hSCARB2 knock‐in mice than that in nonphospho‐Ser/Thr125A (S/T125A) mutants. Notably, phosphorylation‐mimic mutations caused deleterious changes in 2A^pro^ catalytic function (S/T125D/E) and in viral propagation (S125D). Crystal structure simulation analysis showed that Ser125 phosphorylation in EV‐A71 2A^pro^ enabled catalytic Cys to adopt an optimal conformation in the catalytic triad His‐Asp‐Cys, which enhances 2A^pro^ proteolysis. Therefore, we are the first to report Ser/Thr125 phosphorylation of 2A^pro^ increases enteroviral adaptation to the host to ensure enteroviral multiplication, causing pathogenicity. Additionally, weakened viruses containing a S/T125A mutation could be a general strategy to develop attenuated *Enterovirus* vaccines.

## 1 INTRODUCTION

Enteroviruses (EVs), belonging to the *Picornaviridae* and *Enterovirus*, include *Enterovirus A* to *L* and *Rhinovirus A to C* (RV A to C), with more than 300 virus types characterized by phylogenetic clustering.^1^, ^2^ Certain EVs infect humans and cause one billion cases per year, with clinical symptoms ranging from mild to severe or even lethal: hand–foot–mouth disease caused by enterovirus A71 (EV‐A71) and coxsackievirus (CV) A2, 6, 8, 10, 16; respiratory illness caused by enterovirus D68 (EV‐D68) and RVs; poliomyelitis, aseptic meningitis, and encephalitis caused by poliovirus (PV) and EV‐A71; and myocarditis caused by coxsackievirus B3 (CVB3).^3^ Notably, EV‐D68 and EV‐A71 are the common causes of meningitis worldwide and are involved in other severe neurological conditions, such as acute flaccid myelitis and encephalitis, leaving many who survive with lasting paralysis and other disabilities.^3^, ^4^ Further, CVB3 is responsible for acute and chronic diseases, including myocarditis^5^ and type I diabetes.^6^ Nevertheless, no effective treatment or vaccine is currently available for a majority of them.^3^, ^7^ Therefore, further clarification of the molecular mechanisms underlying enteroviral infections is paramount.

Notably, EVs share a highly conserved single‐stranded RNA genome with a 5′‐untranslated region (5′‐UTR), two overlapping open reading frames (ORF1 and ORF2), and 3′‐UTR.^1^, ^8^, ^9^ Also, EVs use internal ribosomal entry site (IRES)‐dependent translation, which is distinct from cellular cap‐dependent translation, thereby strengthening their competitive advantage.^10^, ^11^, ^12^ This activity is the result of the well‐characterized cleavage of eukaryotic initiation factor 4GI (eIF4GI) by enteroviral 2A proteinase (2A^pro^), the proteolytic activity of which relies on the catalytic triad His‐Asp‐Cys, a highly conserved sequence motif in EV proteases.^11^, ^13^, ^14^, ^15^ Cleaved eIF4GI favors viral IRES (vIRES) translation initiation but inhibits cellular cap‐dependent translation initiation. Thus, eIF4GI hydrolysis by 2A^pro^ is the primary event that establishes cellular conditions favor vIRES‐dependent translation and viral proliferation.^14^, ^15^ The proteolytic function of 2A^pro^ also contributes to evasion of the innate immune system by targeting melanoma differentiation‐associated gene 5 (MDA5),^16^ mitochondrial antiviral signaling protein (MAVS),^17^ and type I interferon receptor 1 (IFNAR1)^18^, ^19^ and by promoting apoptosis,^20^ thereby creating a conducive condition for viral survival.

The roles of 2A^pro^ in viral survival and pathogenesis have been well established. Recent studies have shown that host phosphorylation pathways such as Raf/MEK/ERK,^20^, ^21^, ^22^, ^23^ PTEN–mTOR,^24^ PKR,^25^ and Jak/STAT^26^ are involved in enteroviral replication and pathogenesis. Our previous studies showed that the inhibition of extracellular signal‐regulated kinase (ERK), a most important Ser/Thr protein kinases, can block the proteolytic strategy of enteroviral 2A^pro^,^27^, ^28^ suggesting that the phosphorylation events associated with enteroviral 2A^pro^ function as a viral adaptation strategy within infected host cells. In this study, we sought to reveal a potential phosphorylation modification as an adaptive mechanism underlying the regulation of 2A^pro^‐mediated proteolysis and viral survival.

## 2 MATERIALS AND METHODS

### 2.1 Virus and cell culture

EV‐A71 BC08 (accession no. JQ514785) and CVB3 Woodruff (accession no. U57056) were propagated in cells at various multiplicity of infection (MOI). Rhabdomyosarcoma (RD), human embryonic kidney (HEK) 293, and HeLa cells were grown in Dulbecco's modified Eagle's medium (DMEM; Thermo Scientific Hyclone) supplemented with 2% or 10% heat‐inactivated fetal bovine serum (FBS; Life Technologies Gibco) at 37°C in a 5% CO_2_ humidified incubator.

### 2.2 Inhibitors and agonists of the ERK pathway

Highly selective inhibitors of MEK1/2 U0126 (Pierce) and the multikinase inhibitor sorafenib (SF; Sigma‐Aldrich) were used at final concentrations of 30 or 2 μM, which were noncytotoxic in both cases. The ERK‐specific agonist epidermal growth factor (EGF) (PeproTech) was used at a final concentration of 10 ng/ml.

### 2.3 Expression plasmids and site‐directed mutations

**Figure 1:**
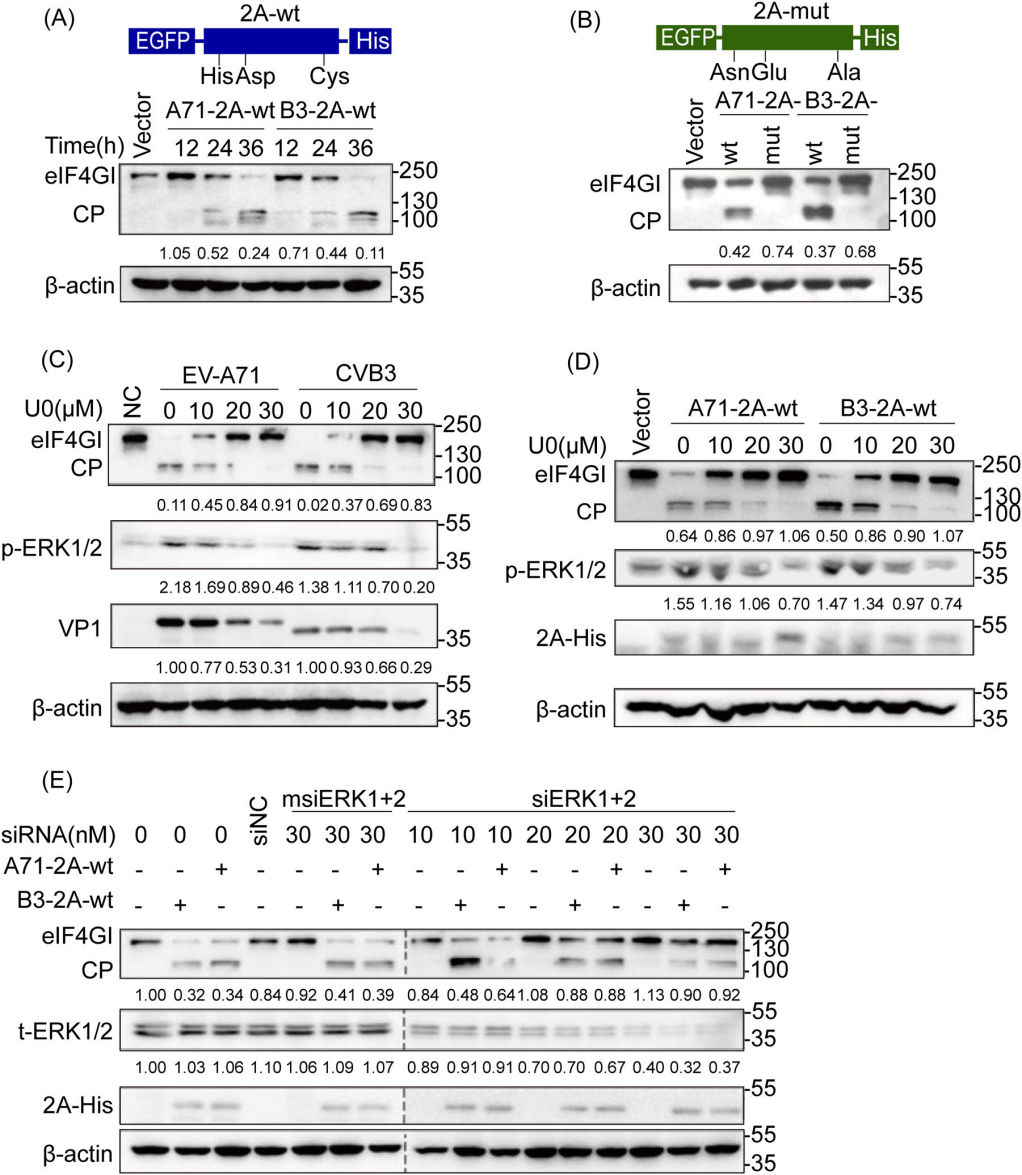
Effect of ERK phosphorylation on 2A‐mediated eIF4GI cleavage of EV‐A71 and CVB3. (A) Human embryonic kidney (HEK) 293 cells transfected with 2 μg of A71‐2A‐wt, B3‐2A‐wt, or vector as a control were harvested and lysed at 12, 24, and 36 h posttransfection for WB analysis. (B) HEK 293 cells transfected with A71‐2A‐mut, B3‐2A‐mut, or vector were harvested and lysed at 24 h posttransfection for WB analysis. (C) RD cells preinfected with 1 multiplicity of infection of EV‐A71 or CVB3 for 2 h were incubated with 10, 20, and 30 μmol/L U0 for 24 h and lysed for WB analysis. (D) HEK 293 cells transfected with 2 μg A71‐2A‐wt or B3‐2A‐wt were incubated with U0 at different concentrations and lysed for WB analysis. (E) HEK 293 cells pretransfected with siNC, *msiERK1*+*2*, or *siERK1*+*2* for 12 h to knock down ERK1/2 were transfected with A71‐2A‐wt or B3‐2A‐wt and then lysed for WB analysis. 50 μg protein was used for 2A's (EGFP‐2A‐His) examination. The ratio of eIF4GI (or VP1/p‐ERKs/t‐ERKs) to the respective vector or NC group signals shown in Arabic numeric in (A–E) was determined using densitometric scanning. The experiments were repeated three times. A71‐2A‐wt, eukaryotic protein expression plasmid of EV‐A71 2A^pro^ wild‐type; B3‐2A‐wt, eukaryotic protein expression plasmid of CVB3‐2A^pro^ wild‐type; vector, eukaryotic protein expression empty plasmid; A71‐2A‐mut, plasmid A71‐2A^pro^ catalytic triad mutant; B3‐2A‐mut, plasmid CVB3‐2A^pro^ catalytic triad mutant. CP, cleavage products; CVB3, coxsackievirus B3; eIF4GⅠ, eukaryotic translation initiation factor 4*γ* I; ERK, extracellular‐signal regulated kinase; EV‐A71, enterovirus A71; RD, rhabdomyosarcoma; U0, U0126; WB, western blotting.

The plasmids pcDNA3.1 and pET21a were used to construct the eukaryotic protein expression plasmid pcDNA3.1 EGFP‐EV‐A71 2A^pro^‐His (A71‐2A‐wt) and prokaryotic protein expression plasmid pET21a‐EV‐A71 P1‐2A^pro^ (P1‐2A‐wt) by amplifying 2A^pro^ and P1‐2A^pro^ from the genome of EV‐A71 BC08, subcloning into pcDNA3.1‐EGFP and PET21a predigested with HindIII/XbaI and BamHI/XhoI, respectively, and introducing a His tag into the C‐terminus of EV‐A71 2A^pro^. The other 2A^pro^ set eukaryotic expression plasmids in Figure 1 (B3‐2A‐wt) and six were constructed as described above, replacing the EV‐A71 2A^pro^ sequences with the indicated enteroviral 2A^pro^ sequences derived from GenBank (CVB3, U57056; PV, V01149; EV‐D68, AY426531; HRV, FJ445111).

EV‐A71 2A^pro^ catalytic triad His‐Asp‐Cys mutated plasmids (A71‐2A‐mut) harboring changes within the active sites of the triad (His21Asn, Asp39Glu, and Cys110Ala) derived from A71‐2A‐wt was generated by site‐directed mutations. A set of Ser point‐mutant plasmids were generated using the same strategy. All polymerase chain reactions (PCRs) were performed using the I‐5 Hi‐Fi DNA Polymerase kit (iCloning), and plasmid assembly reactions were performed under Gibson assembly reaction conditions (NEB).

The full and abbreviated names of plasmids are shown in Supporting Information: Table 1. All primers used are listed in Supporting Information: Table 2.

### 2.4 Small interfering RNAs (siRNAs) and transient transfection

siRNAs (*siERK1+2*) were used to knockdown both *ERK1* and *ERK2*. Mutant siRNAs (*msiERK1*+2) and control siRNAs (siNC) were simultaneously applied. The siRNA sequences are listed in Supporting Information: Table 3 (GenePharma Co. Ltd.). Also, siRNA transfection was performed according to the instruction using Lipofectamine 2000 (Invitrogen).

### 2.5 Western blotting (WB)

Total cell lysates were obtained using RIPA buffer (P0013; Beyotime). WB was conducted as previously described.^28^ The primary antibodies used are as follows: phosphothreonine (p‐Thr, ab9337; Abcam), phosphoserine (p‐Ser, ab9332; Abcam), eIF4GI (2858s; Cell Signaling Technology), MDA5 (5321s; Cell Signaling Technology), GFP (2955s; Cell Signaling Technology), VP1 (PAB7631‐D01P; Abnova), VP1(GTX633390; CeneTex), His (Biodragon, B1004), total ERK1/2 (t‐ERK1/2, 9102s; Cell Signaling Technology), phospho‐ERK1/2 (p‐ERK1/2, 9106s), and β‐actin (BS6007 M; BioWorld). Specific bands were visualized using enhanced chemiluminescence. The grayscale intensity of each WB band was quantified using ImageJ (version 1.52a).

### 2.6 His pull‐down assays

Cells were harvested and lysed in buffer B (8 M urea, 100 mM NaH_2_PO_4_, 10 mM Tris pH 8.0, 20 mM imidazole). Proteins were precipitated with Ni‐NTA agarose (Qiagen) and washed four times with buffer C (8 M urea, 100 mM NaH_2_PO_4_, 10 mM Tris‐HCl, pH 6.3), eluted with buffer E (8 M urea, 100 mM NaH_2_PO_4_, 10 mM Tris‐HCl, pH 4.0; 250 mM imidazole) and measured using WB.

### 2.7 Co‐immunoprecipitation

Cells were harvested and lysed with NETENG‐400 buffer (400 mM NaCl, 20 mM Tris‐HCl, pH 7.4, 0.1% Nonidet P‐40, 0.5 mM ethylene diamine tetraacetic acid, 1.5 mM MgCl_2_, 10% glycerol) containing cocktail (5892791001; Roche). The lysates were diluted to a final NaCl concentration of 150 mM and mixed with Protein G beads (BDTL0003; Biodragon) and His or IgG antibody (sc‐2025; Santa Cruz Biotechnology) and then rotated at 4°C overnight. The samples were eluted with 100 mM glycine (pH 2.5), neutralized by 1 M Tris‐HCl (pH 8.5), and then analyzed using immunoblotting.

### 2.8 In vitro phosphorylation assay

HEK 293 cells transfected with plasmids encoding wild‐type (wt) ERK2 and dead ERK2, starved overnight, stimulated with EGF (10 ng/ml), and then harvested and lysed. The resultant supernatants were mixed with glutathione beads, shaken, and centrifuged. The supernatant was discarded, and the collected beads were eluted with 50 mM glutathione at 4°C overnight and then purified using Protein G beads and anti‐His antibodies. The mixture containing purified kinase protein, target proteins, ATP, and the appropriate kinase buffer was incubated at 37°C for 10 min and detected using WB.

### 2.9 Protein expression in the prokaryotic system

The constructed plasmid pET21a P1‐2A^pro^ set (P1‐2A‐wt, P1‐2A‐S125A, and P1‐2A‐S125D in Figure 3E) was transformed into *Escherichia coli* Rosetta and the transformants were cultured in Luria–Bertani medium containing 100 μg/ml ampicillin and then induced by IPTG (Isopropyl β‐d‐Thiogalactoside, 1.0 mm). The cells were harvested and resuspended in lysis buffer, treated with lysozyme (Sigma‐Aldrich), and sonicated on ice. Debris was removed by centrifugation and the supernatant was purified using a B‐PER 6×His fusion protein purification kit (Thermo Scientific Pierce), according to the manufacturer's instructions.

### 2.10 Protein expression in rabbit reticulocyte lysates (RRL)

The plasmid pBR322‐5′UTR‐P1‐2A‐3′UTR (pIRES P1‐2A‐wt in Figure 3F) was constructed subcloned with a Lightening Cloning Kit (BDIT0014; Biodragon). Further, pIRES P1‐2A‐S125A and pIRES P1‐2A‐S125D, derived from pIRES P1‐2A‐wt, were generated using site‐directed mutation. In vitro transcription was used to synthesize RNAs from the corresponding recombinant DNA templates (p1300; Promega). Protein was overexpressed according to the instructions of the RRL System (L4151; Promega).

### 2.11 Infectious clone and rescued virus

The plasmids pBR322‐EV‐A71 derived from the genomic RNAs of EV‐A71 BC08 strain (from Qing Xiong) were used to obtain the pBR322‐EV‐A71 mutants either with S125A or S125D by site‐point mutation (Supporting Information: Table 4, Supporting Information: Figure 1) using the Fast Mutagenesis System kit (TransGen Biotech) and then in vitro transcribed to RNAs (A71 RNA wt, A71 RNA S125A, A71 RNA S125D) by RiboMAX Large Scale RNA Production System‐T7 (Promega). Wt genomic RNAs (CVB3 RNA wt), T125A genomic RNAs (CVB3 RNA T125A), and T125E genomic RNAs (CVB3 RNA T125E) of CVB3 Woodruff were obtained using the same methods. The progeny‐rescued viruses were recovered by transfecting RNA transcripts into RD cells.

### Ethics statement and animal studies

The experimental mice used in the study were housed and strictly handled in accordance with the institutional guidelines (National Institute for Food and Drug Control, NIFDC) for animal care and use. The study protocol was approved by the Institutional Animal Care and Use Committee of NIFDC. The in vivo animal experiments were based on a previous study. For virulence, groups of 4‐week‐old human SCARB2 knock‐in mice (hSCARB2 mice) were intravenously inoculated with the wt EV‐A71 Isehara strain (Ise‐EV‐A71 wt, *n* = 7), obtained from Prof. Satoshi Koike, and the corresponding Ise‐EV‐A71 S125A (*n* = 7) at 10^7^ TCID50/ml. For the control, DMEM was intravenously administered to hSCARB2 mice (*n* = 7). The animals were monitored daily for 14 days after inoculation, including changes in body weight, clinical scores, and survival rates. Clinical scores were based on a record chart of clinical signs (Supporting Information: Table 5). Mice found in the moribund condition were euthanized and scored as dead.

### 2.12 Histopathologic and immunohistochemical staining

Mouse tissues were collected and fixed in 10% neutral buffered formalin, embedded in paraffin, and sectioned (3 μm). For histopathological examination, the tissue sections with DEME, wt, and A‐mutated viruses were stained with HE. The two‐step Envision method was used to perform immunohistochemical staining on the tissue sections. Horseradish peroxidase‐conjugated goat anti‐mouse secondary antibody was used after anti‐EV‐A71 antibody (MAB979; Millipore, 1:5000 dilution) or isotype IgG (control). The cells were examined using confocal microscopy (LSM 700; Zeiss).

### 2.13 Quantitation of viral loads in different organs

Tissues were ground in liquid nitrogen and the total RNA was extracted with TRIzol reagent (Life Technologies Invitrogen). Reverse transcribed to generate cDNA in accordance with the instructions using the RevertAid First Strand cDNA Synthesis Kit (Thermo Scientific). The viral loads were detected by quantitative reverse‐transcription polymerase chain reaction (RT‐qPCR) using primers 5′‐GCGATGAGAGTATGATTGAGACACG‐3′ and 5′‐CCTGTTATGTCTATGTCCCAATTTGC‐3′.

### 2.14 Alignment and phylogenetic analysis

The 666 amino acid sequence sets of EV‐A71 2A^pro^ from GenBank were aligned using MEGA5.0. A phylogenetic tree based on alignment of the indicated enteroviral 2A^pro^ sequences from GenBank was constructed using the neighbor‐joining method. Numbers at the nodes represent bootstrap values.

### 2.15 Indirect fluorescence assay

Cells transfected with plasmids or RNA transcripts were washed, fixed with 4% paraformaldehyde, permeabilized with 0.1% Triton X‐100 and subsequently blocked. Samples were incubated with primary antibody, anti‐p‐ERK (4377s; Cell Signaling Technology), or anti‐VP1 (MAB979; Merck Millipore) at a dilution of 1:200, then with secondary antibodies (1:500), and finally with DAPI for 5 min. Cells were examined by confocal microscopy (Leica) using Leica fluorescence microscope (Leica DFC 365 FX).

### 2.16 Absolute RT‐qPCR

The supernatant and cell lysates were treated with 100 μg/ml RNase A to eliminate naked viral RNAs, and RNAs were extracted using the EasyPure Viral RNA Kit (Trans). Reverse transcription was performed using the RevertAid First Strand cDNA Synthesis Kit (Thermo Scientific). Subsequently, EV‐A71 virions were analyzed using absolute RT‐qPCR technique with the ABI 7500 real‐time PCR system (Applied Biosystems) and the QuantiTect SYBR Green RT‐PCR Kit (Qiagen) and specific forward and reverse primers targeting the conserved region of *VP1*.

### 2.17 Molecular dynamics simulations

Molecular dynamics simulations were performed using the AMBER 11 molecular simulation package (AMBER 11 Users' Manual, University of California, San Francisco, 2010). To obtain the molecular mechanical parameters for phosphorylated serine, ab initio quantum chemical methods were performed using the Gaussian 09 program (Revision A.1; Gaussian, Inc., 2009). The final conformations of the complexes were produced from the 1000 steps of the minimized averaged structure of the last 10 ns of molecular dynamics.

### 2.18 Statistical analysis

All diagrams were generated using GraphPad Prism v7.0. Data were obtained from at least three independent experiments and were presented as mean ± SD. Differences were analyzed using the Student's *t* test. *p < 0.05, **p < 0.01, and ***p < 0.001 were considered statistically significant.

## 3 RESULTS

### 3.1 ERK phosphorylation favors eIF4GI cleavage by EV‐A71 and CVB3 2A^pro^

Based on our previous work,^27^, ^28^ we further confirmed better cleavage of eIF4GI in the cells transfected with plasmids expressing wt EV‐A71 2A^pro^ and CVB3 2A^pro^, designated as A71‐2A‐wt and B3‐2A‐wt (Figure 1A), but not in cells transfected with the respective His‐Asp‐Cys mutants, corresponding to A71‐2A‐mut and B3‐2A‐mut (Figure 1B). Further, the cleavage process was blocked in a dose‐dependent manner by U0126, a highly selective inhibitor of MEK1/2, in cells infected with wt EV‐A71 BC08 or CVB3 Woodruff (Figure 1C) or by both U0126 and siRNA‐mediated knockdown of ERKs in cells transfected with A71‐2A‐wt or B3‐2A‐wt (Figure 1D,E). The results obtained with both perturbing ERK activity and disturbing 2A^pro^ cleavage activity demonstrated that ERK phosphorylation is required for the proteolytic activity of enteroviral 2A^pro^ and suggests the possibility and importance of 2A^pro^ phosphorylation in its function and viral survival.

### 3.2 Phosphorylation and phosphorylation site of EV‐A71 2A^pro^ identification

To further characterize the phosphorylation of enteroviral 2A^pro^, we overexpressed A71‐2A‐mut for two reasons: first, to overcome the weak low expression level of EV‐A71 2A^pro^ in cells due to viral 2A^pro^‐associated shutdown of cap‐dependent translation (Supporting Information: Figure 2A,B); second, phosphorylation modifications should not occur on the 2A catalytic triad His‐Asp‐Cys^29^ due to the Ser/Thr substrate preferences of ERK.^30^ Accordingly, we used A71‐2A‐mut to identify the potential phosphorylation site(s) in EV‐A71 2A^pro^. Notably, ERKs were activated upon overexpression of A71‐2A‐wt or A71‐2A‐mut (Supporting Information: Figure 2C,D).

**Figure 2:**
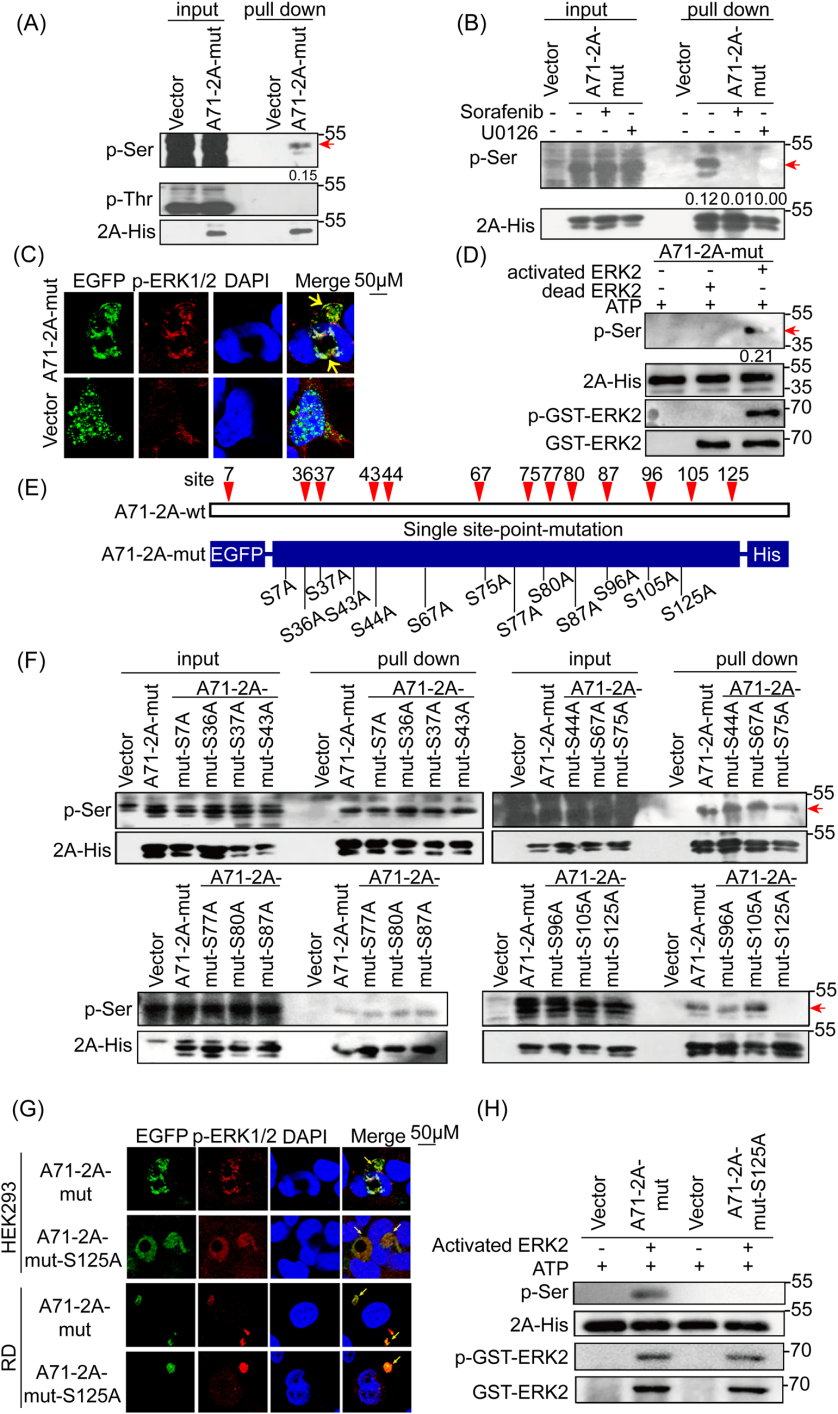
Identification of EV‐A71 2A phosphorylation at Ser125 by activated ERK1/2. (A) HEK 293 cells transfected with 2 μg of A71‐2A‐mut or vector were lysed at 48 h posttransfection and collected by pull down for WB analysis. (B) HEK 293 cells transfected with A71‐2A‐mut or vector were harvested with U0126 (30 μM) or Sorafenib (2 μM) for 48 h and collected by His pull‐down for WB analysis. (C) HEK 293 cells transfected with A71‐2A‐mut or vector were fixed in 4% paraformaldehyde, permeabilized with 0.1% Triton X‐100, and stained with anti‐p‐ERK (red); A71‐2A‐mut was stained with EGFP fusion protein (green), and nuclear DNA with DAPI (blue). The cells were observed using confocal microscopy (LSM 700; Zeiss). Scale bar = 50 μm. Yellow arrows indicate target proteins. (D) HEK 293 cells transfected with A71‐2A‐mut and GST‐ERK2 were lysed after 48 h, and proteins were purified and mixed with buffer with activated ERK2 or kinase‐dead ERK2 in vitro. (E) Distribution of 13 individual Ser residues on A71‐2A. (F) HEK 293 cells transfected with S‐to‐A mutations were collected after 48 h for WB analysis. (G) HEK 293 and RD cells transfected with A71‐2A‐mut or A71‐2A‐mut‐S125A for 24 h were fixed, permeabilized, and stained, as shown in (C), and were observed using confocal microscopy. Scale bar = 10 μm. Yellow arrows indicate target protein interactions. (H) Phosphorylation in vitro was conducted as shown in (D) with either A71‐2A‐mut or A71‐2A‐mut‐S125A. The ratios of the p‐Ser to the respective 2A‐His signals shown in Arabic numbers in (A, B, D) were determined using densitometric scanning. The experiments were repeated three times. A71‐2A‐mut, plasmid A71‐2A^pro^ catalytic triad mutant; A71‐2A‐wt, eukaryotic protein expression plasmid of EV‐A71 2A^pro^ wild‐type; vector, eukaryotic protein expression empty plasmid; GST‐ERK2, eukaryotic expression plasmid for the fusion proteins ERK2 and GST. A71‐2A‐mut‐S125A, plasmids carrying the 125 site mutation of the A71‐2A^pro^ catalytic triad mutant. ERK, extracellular‐signal regulated kinase; EV‐A71, enterovirus A71; HEK 293, human embryonic kidney 293; RD, rhabdomyosarcoma; WB, western blotting.

Our results showed that overexpressed A71‐2A‐mut was phosphorylated on Ser, but not Thr, with approximately 15% (p‐Ser/2A‐His) of the total protein (Figure 2A); no phosphorylation was detected on the EGFP (Enhanced Green Fluorescent Protein) tag using the His pull‐down assay (Supporting Information: Figure 2C). In addition, the inhibition of ERK1/2 activation by U0126 (U0) and SF prevented A71‐2A‐mut phosphorylation (Figure 2B, Supporting Information: Figure 2E), suggesting that A71‐2A‐mut was phosphorylated by p‐ERK1/2. Furthermore, the colocalization of p‐ERK1/2 with A71‐2A‐mut (Figure 2C) and the in vitro phosphorylation of A71‐2A‐mut by active ERK2 (pGST‐ERK2) (Figure 2D) provided further evidence that A71‐2A‐mut was phosphorylated. Thus, our results indicate that the Ser residue of EV‐A71 2A^pro^ is phosphorylated by ERK1/2. To further narrow down the potential phosphor‐Ser site(s), we identified a total of 13 conserved serine sites in an alignment of 666 EV‐A71 2A sequences obtained from GenBank (data not shown). Based on this, a series of A71‐2A‐mut variants with Ser (S) to Ala (A) point mutations were generated to determine the phosphorylation site(s) (Figure 2E, Supporting Information: Figure 2F). The data showed that only the S125A mutation inhibited 2A^pro^ phosphorylation, as determined using His pull‐down assay, followed by immunoblotting with anti‐S/T panphosphorylation antibodies (Figure 2F). Consistent with the above conclusions, A71‐2A‐mut‐S125A colocalized with p‐ERK1/2 in the cytoplasm (Figure 2G) and completely lost the phosphorylation signal in cell‐free experiments (Figure 2H), confirming that Ser125 was indispensable for A71‐2A‐wt phosphorylation. Taken together, these data demonstrate that phosphorylation of EV‐A71 2A^pro^ at Ser125 is mediated by activated ERK1/2.

### 3.3 Ser125 phosphorylation favors EV‐A71 2A^pro^ trans‐cleavage activity and viral propagation

**Figure 3:**
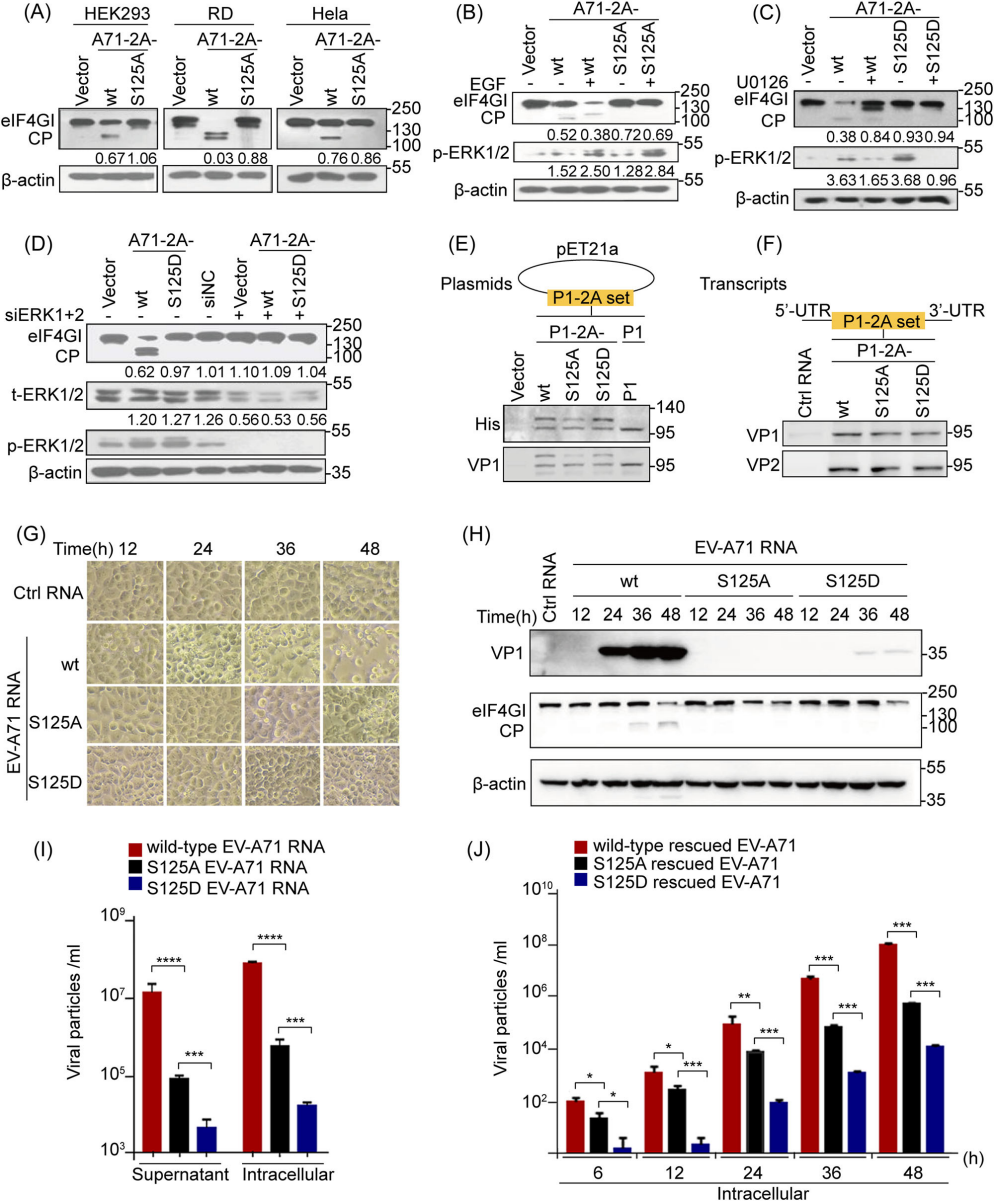
Ser125 phosphorylation favors EV‐A71 2A trans‐cleavage activity and viral propagation. (A) HEK 293 cells, RD, and HeLa cells transfected with A71‐2A‐wt and A71‐2A‐S125A were collected and lysed at 48 h for WB analysis. (B) HEK 293 cells transfected with A71‐2A‐wt and A71‐2A‐S125A were treated with epidermal growth Factor (10 ng/ml) for 24 h and lysed for WB analysis. (C) HEK 293 cells transfected with A71‐2A‐wt and A71‐2A‐S125D were treated with U0126 (30 μM) for 24 h and lysed for WB analysis. (D) HEK 293 cells pretransfected with or without siERK1+2 (30 nM) for 12 h were treated with A71‐2A‐wt or A71‐2A‐S125D for 24 h and lysed for WB analysis. The ratio of the eIFGI (or p‐ERKs/t‐ERKs) to the respective vector group signals shown in Arabic numbers in (A–D) was determined using densitometric scanning. The experiments were repeated three times. (E, F) Cis‐cleavage activity by wt, S125A, or S125D mutation of P1‐2A protein was observed from the pET21a‐P1‐2A set in the bacterial system (E) and RNAs in vitro transcribed from the pIRES P1‐2A set in the eukaryotic system (F). (G, H) wt, S125A, and S125D EV‐A71 genomic RNAs transcribed in vitro were transfected at 3 μg into RD cells. Cytopathic effects were observed at 12, 24, 36, and 48 h (×200 magnification) (G), and cell lysis was detected using WB (H). (I) Intracellular levels of viral particles and the particle levels in the supernatant were measured at 48 h postinfection using absolute. (J) Intracellular viral particles were measured at different time points in RD cells infected with rescued progeny viruses (MOI = 1) from wt, S125A, and S125D EV‐A71 RNA transcripts. Data are presented as mean ± SD (*n* = 3). *p < 0.05, **p < 0.01, and ***p < 0.001 versus respective controls, as determined by Student's *t* test. ERK, extracellular‐signal regulated kinase; EV‐A71, enterovirus A71; HEK 293, human embryonic kidney 293; MOI, multiplicity of infection; RT‐qPCR, reverse‐transcription polymerase chain reaction; WB, western blotting.

To determine the importance of Ser125 phosphorylation in the proteolytic activity of EV‐A71 2A^pro^, we assessed the well‐characterized trans‐cleavage of eIF4GI in cells and cis‐cleavage of the P1‐2A protein using a set of 125 site point mutations to understand the presumed phospho‐deficient (125A) and phosphorylation‐mimic (125D) status for viral 2A lytic activity. We found that A71‐2A‐S125A exhibited weaker eIF4GI cleavage, with or without EGF, compared with that of A71‐2A‐wt (Figure 3A,B, Supporting Information: Figure 3A). Notably, the phosphorylation mimic mutant A71‐2A‐S125D polished the trans‐cleavage activity of eIF4GI treated with or without U0126 or specific siRNAs targeting ERKs (Figure 3C,D). Furthermore, neither the S125A nor S125D mutants impaired cis‐cleavage activity, as verified by the P1‐2A construct in the bacterial (Figure 3E) and eukaryotic systems (RRL) (Figure 3F), similar to that of A71‐2A‐wt (Supporting Information: Figure 3B). Therefore, these results emphasize the critical role of Ser125 phosphorylation, but not Ser125 mutations, in the efficient cleavage of A71‐2A^pro^, mainly by enhancing trans‐cleavage activity.

However, to elucidate the role of Ser125 phosphorylation at 2A^pro^ in viral replication, cells were transfected with the nonphospho‐S125A genomic RNA transcript of EV‐A71 (A71‐RNA‐S125A) that showed a much weaker cytopathic effect (Figure 3G), lower VP1 protein levels (Figure 3H, Supporting Information: Figure 3C), and fewer progeny viruses by 100‐fold (Figure 3I) than those transfected with the wt genomic RNA transcript of EV‐ A71 (A71‐RNA‐wt). To illustrate the characteristics of phosphorylation in the viral replication cycle, application of S125A‐rescued EV‐A71 (MOI of 1) resulted in delayed one‐step growth dynamics relative to wt‐rescued EV‐A71 (Figure 3J). Thus, the S/T125A mutation attenuated viral replication and reproduction (Figure 3G–J and Supporting Information: Figure 3C). Next, the phosphorylation‐mimic mutation (S125D) inhibited viral production (Figure 3G–J, Supporting Information: Figure 3C) and caused gene polymorphisms in the second and subsequent generations of rescued S125D viruses in cells infected with the first generation of rescued S125D viruses (Supporting Information: Figure 3D), suggesting that the mimic hyperphosphorylation site is unstable and unsuitable for enteroviral survival. In summary, Ser125 phosphorylation guarantees the trans‐cleavage of 2A^pro^ and viral multiplication.

### 3.4 Ser125 phosphorylation favors EV‐A71 pathogenesis

**Figure 4:**
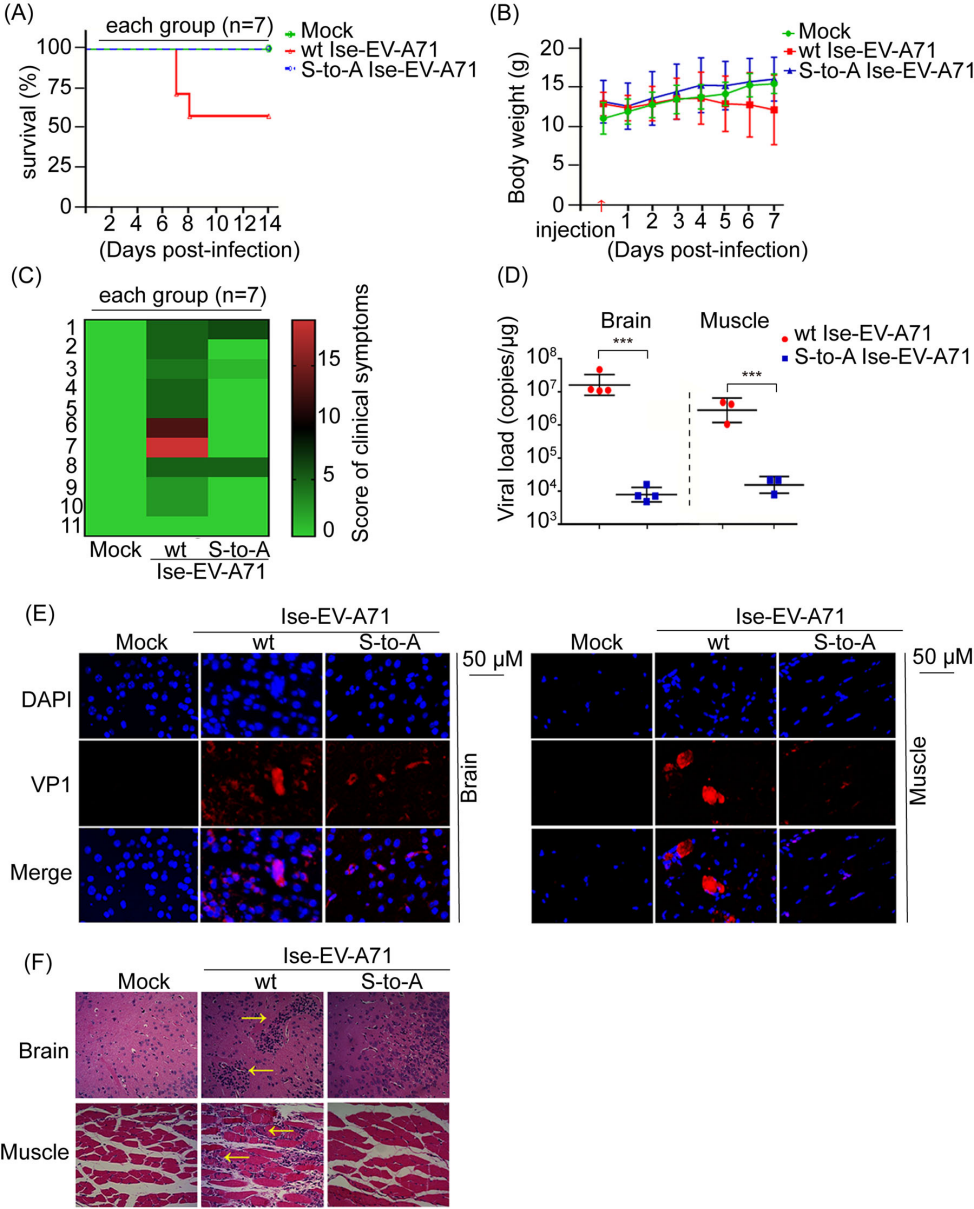
2A Ser125 phosphorylation favors EV‐A71 pathogenesis. (A) Survival rate of 4‐week‐old hSCARB2 knock‐in mice (*n* = 7) intravenously inoculated with wt and S125A EV‐A71 Isehara (Ise‐EV‐A71) (10^7^ TCID50/ml). (B, C) Body weight and scores of clinical symptoms of infected hSCARB2 knock‐in mice. (D) Viral loads of the hind‐limb skeletal muscle and brain at 7 d.p.i. by absolute RT‐qPCR. Data are presented as mean ± standard deviation (*n* ≥ 3). *** *p* < 0.001 as determined by Student's *t* test. (E, F) Waxed section (3 μm) of brain and hind‐limb muscle at 7 d.p.i. (*n* = 3) for immunofluorescence (LSM700; Zeiss, 50 μm) (E) and hematoxylin and eosin staining (×200). Lymphocytic infiltrations with yellow arrows (F). d.p.i., day postinfection; EV‐A71, enterovirus A71; RT‐qPCR, reverse‐transcription polymerase chain reaction; wt, wild‐type.

To further verify viral pathogenesis in vivo, human SCARB2 knock‐in mice (hSCARB2 mice), a well‐established animal model susceptible to EV‐A71 infection, were intravenously (i.v.) inoculated with the susceptible virus EV‐A71 strain Isehara (Ise‐EV‐A71),^31^ either wt or rescued S125A viruses, and monitored for 2 weeks. The outcome assessment based on survival rate, body weight, and clinical signs revealed that the viral pathogenesis of Ise‐EV‐A71 S125A was significantly reduced compared with that of Ise‐EV‐A71 wt in hSCARB2 mice (Figure 4A–C). In particular, the symptoms of hind‐limb paralysis (5/7 mice) and even death (3/7 mice) in the wt peaked on the 7th day postinfection, whereas none occurred in the S125A virus (Figure 4A–C). Furthermore, the viral loads of the S125A virus were reduced by 1000‐fold (Figure 4D, Supporting Information: Figure 4A,B), and the VP1 fluorescence intensities representing the EV‐A71 antigen level were weak (Figure 4E) in the brain and muscle compared with those in the wt. Hematoxylin and eosin (HE) staining of the wt virus showed more severe tissue damage, characterized by lymphocyte infiltration and partial histiocytosis, compared with that of the S125A virus (Figure 4F). Thus, S125A nonphosphorylation probably impairs viral proliferation, thereby reducing viral virulence. Collectively, our results provide further evidence that Ser125 phosphorylation promptes EV‐A71 propagation and pathogenesis in vivo.

### 3.5 Ser125 phosphorylation maintains structural conformation of catalytic triad in EV‐A71 2A^pro^

**Figure 5:**
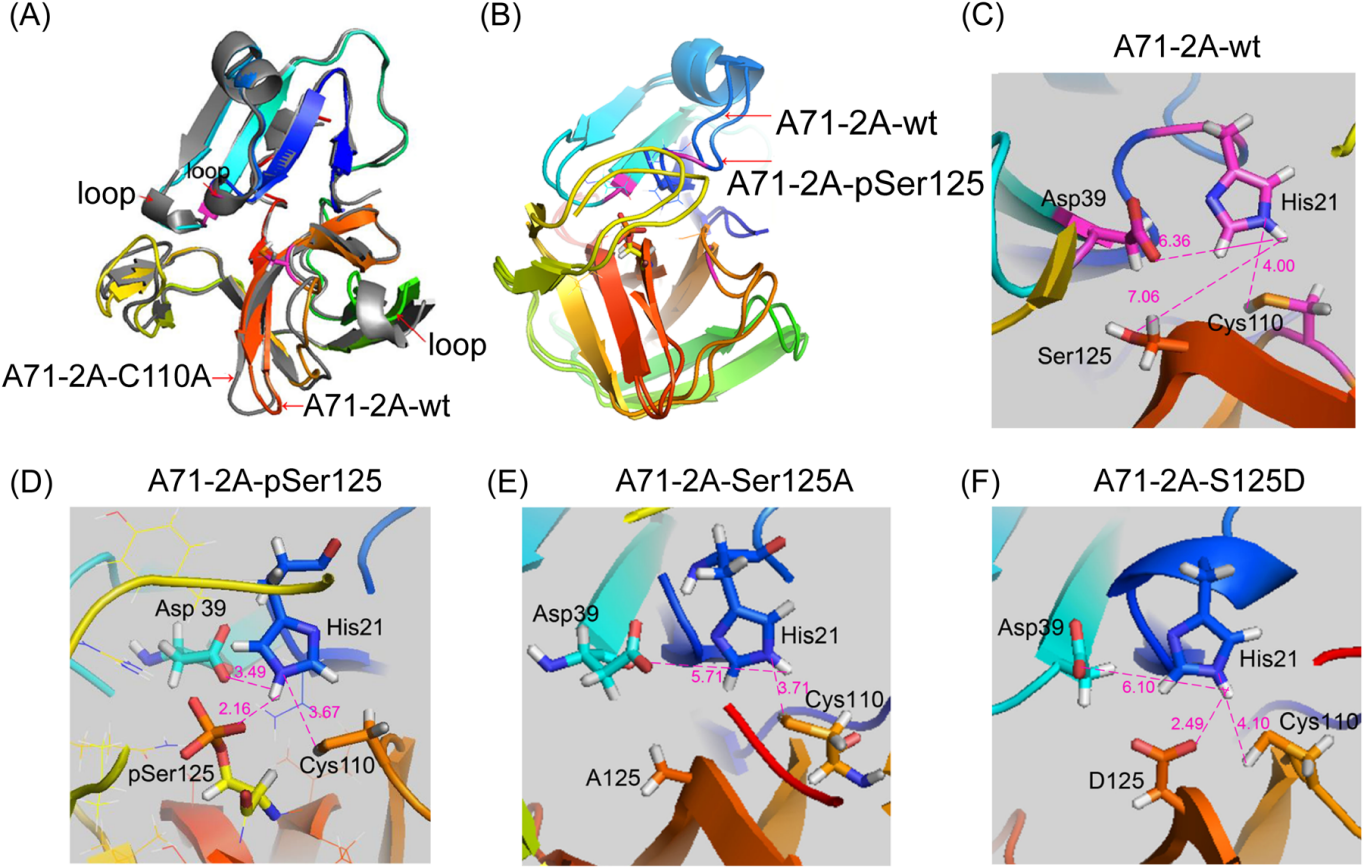
Structural conformation of the EV‐A71 2A set. (A) Molecular dynamics simulation of EV‐A71 2A wild‐type (A71‐2A‐wt) (RMSD of 0.943 Å) derived from A71‐2A‐C110A (gray, PBD code: 4FVD). (B) The crystal structure simulation of A71‐2A‐wt with Ser125 phosphorylation (A71‐2A‐pSer125) on the basis of 4FVD. The RMSD of conformational changes between A71‐2A‐wt and A71‐2A‐pSer125 was only 1.444 Å. (C–F) The crystal structure simulation of S‐to‐A mutants A71‐2A‐S125A and S‐to‐D mutants A71‐2A‐S125D on the basis of 4FVD. The HB networks formed by Ser125 (A71‐2A‐wt), phospho‐Ser125 (A71‐2A‐pSer125), A125 (A71‐2A‐S125A), and D125 (A71‐2A‐S125D) and residues in the catalytic site within the structural conformation. The distances were measured among the Asp‐His‐Cys, phospho‐Ser125, A125, and D125 (magenta lines). EV‐A71, enterovirus A71; RMSD, root mean square deviation.

In general, phosphorylation affects enzyme function in two ways: by altering electronegativity (e.g., D/E residues simulate phosphorylation of S/T) and by altering enzyme conformation. To understand which mechanism was most relevant to Ser125 phosphorylation, dynamic simulation of wt 2A^pro^ (A71‐2A‐wt, Figure 5A–C), wt 2A^pro^ with Ser125 phosphorylation (A71‐2A‐pSer125, Figure 5B,D, Supporting Information: Figure 5), S125A mutation in 2A^pro^ (A71‐2A‐S125A, Figure 5E) and S125D mutation in 2A^pro^ (A71‐2A‐S125D, Figure 5F) were applied to the model of the crystal structure 2A^C110A^ of EV‐A71 with a false substrate.^32^ Enteroviral 2A^pro^ cleavage activity is based on the catalytic triad His‐Asp‐Cys; in particular, the –SH group of Cys attacks the carbonyl carbon of the substrate, and the nitrogen of His accepts the hydrogen from –SH. Dynamic simulation analyses revealed that phosphorylated Ser125 formed a stronger hydrogen bond (HB) network with Asn19, His21, Asp39, Arg93, and Gln95 (Figure 5D), resulting in a shorter His‐Asp‐Cys interaction distance than that in A71‐2A‐wt (Figure 5C). As shown in Figure 5D, the proton hydrogen (H) of –SH from Cys110 that was taken up by the nitrogen (N) of His21 (3.67 Å) caused the –S to attack the carbonyl carbon of the substrate. The HBs between the carbonyl group of Asp39 and H of His21 (3.49 Å) and the phosphate groups of pSer125 and H of His21 (2.16 Å) assisted the N of His21 in accepting proton H. Taken together, these alterations made the N of His21 easily accept the H of –SH from Cys110, facilitating 2A^phospho^ hydrolysis of the substrate by the –SH group. Regarding A71‐2A‐S125A, only the N of His21 united proton H of –SH in Cys110 (3.71 Å), preserving partial capacity and attenuating cleavage activity (Figure 5E). Notably, the carbonyl group of A71‐2A‐S125D accepted and shared proton H with the N of His21 (2.49 Å), reaching a steady state (Figure 5F); thus, the N of His21 could not accept the H of –SH from Cys110, suggesting that the –S group of Cys110 lost the ability to attack the carbonyl carbon of the substrate. Taken together, dynamic simulation analyses confirmed that Ser125 phosphorylation favors 2A^pro^ folding, which benefits EV‐A71 2A^pro^ trans‐cleavage activity.

### 3.6 2A Ser/Thr125 phosphorylation in proteolytic activity is highly conserved in the *Enterovirus*

**Figure 6:**
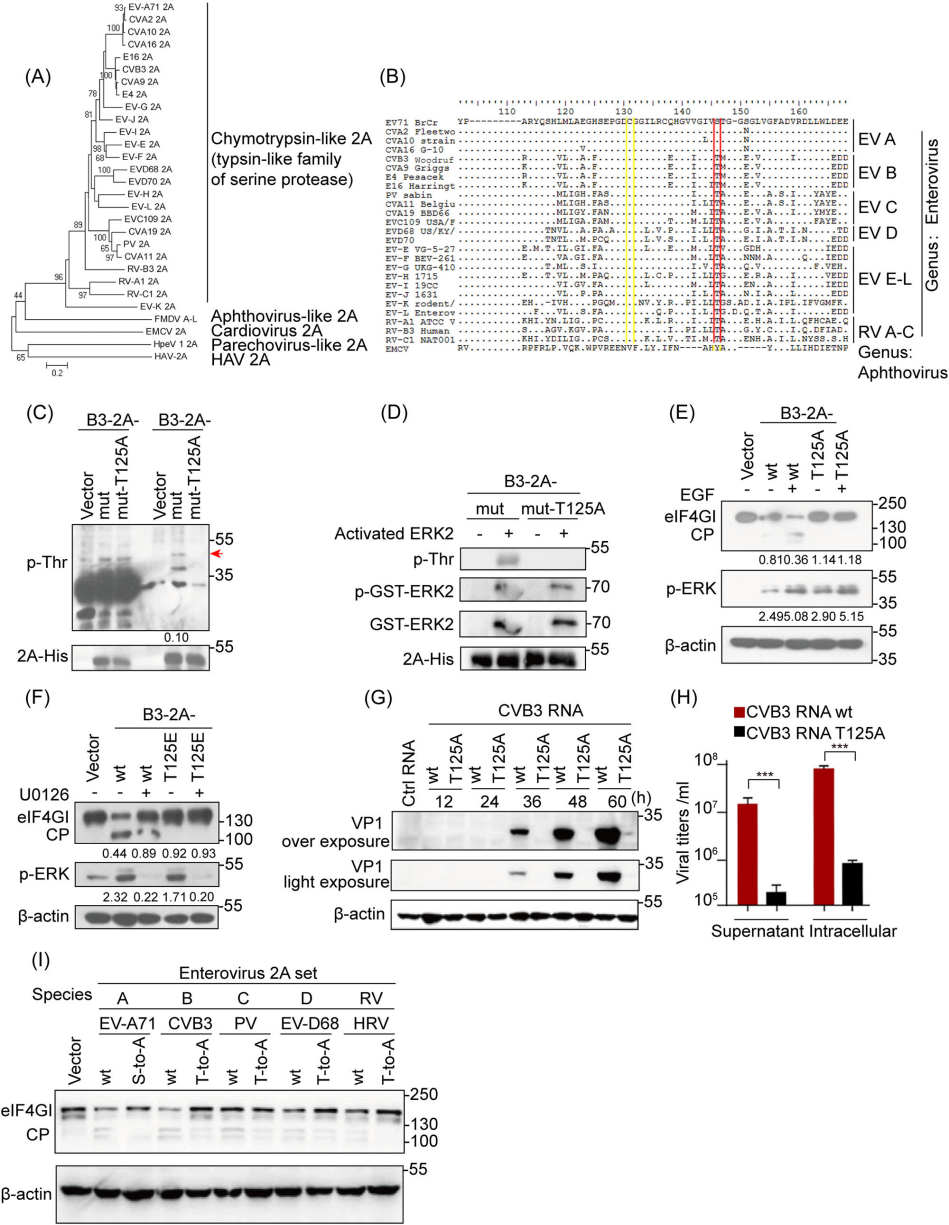
2A phosphorylation at Ser/Thr125 in proteolytic activity is highly conserved in the genus *Enterovirus*. (A) The amino acid sequences of enterovirus 2A protein were obtained from GenBank and constructed into phylogenetic trees using the neighbor‐joining method. The numbers at the nodes represent bootstrap values. HPeV‐1 (parechovirus‐like), FMDV (aptovirus‐like), EMCV (cardiovirus), and HAV (hepatovirus A) were used as controls. (B) Sequences of enterovirus 2A protein of EV A–L and RV A–C were aligned using MEGA (version 5.05). The relevant sequences of Aphthovirus (family *Picornaviridae*) were used as controls. The conserved Ser/Thr125 residues are marked in red. (C) HEK 293 cells transfected with 2 μg of B3‐2A‐mut or vector were lysed at 48 h posttransfection and collected by pull down for WB analysis. The ratio of the p‐Thr to 2A‐His signals shown in Arabic numerals was determined using densitometric scanning. (D) Phosphorylation was conducted in vitro as shown in Figure 2D with either B3‐2A‐mut or B3‐2A‐mut‐T125A. (E, F) HEK 293 cells transfected with B3‐2A‐wt and B3‐2A‐T125A or B3‐2A‐T125E were treated with EGF (10 ng/ml) (E) or U0126 (30 μM) (F) for 24 h and lysed for WB analysis. The ratio of the eIF4GI (or p‐ERK) to the respective vector group signals shown in Arabic numerals in (E, F) was determined using densitometric scanning. The experiments were repeated three times. (G, H) RD cells transfected with 3 μg wt or T125A CVB3 genomic RNAs transcribed in vitro were lysed for WB analysis (G), and intracellular levels of viral particles and the particle levels in the supernatant were measured at 48 h post infection. using absolute RT‐qPCR (H). (I) HEK 293 cells transfected with 2 μg plasmid of S/T‐to‐A on 2 A of EV‐A71, CVB3, PV, EV‐D68, and HRV were harvested and lysed at 24 h for WB analysis. Data are presented as mean ± SD (*n* ≥ 3). *p < 0.05, **p < 0.01, ***p < 0.001 as determined by Student's *t* test. CVB3, coxsackievirus B3; EGF, epidermal growth factor; HEK 293, human embryonic kidney 293; NJ method, neighbor‐joining method; RT‐qPCR, reverse‐transcription polymerase chain reaction; WB, western blotting.

The 2A^pro^ homologs in *Picornaviridae* is divided into five types according to their structures and functions.^1^, ^15^ First, the phylogenetic analysis of the representative full‐length amino acid sequences of 2A from species A to L of *Enterovirus*, as well as RV A to C from GenBank (Figure 6A), demonstrated high degree of similarity with those of common evolutionary ancestry, suggesting that enteroviral 2A proteins belong to a trypsin‐like family.^33^ Notably, sequence alignment identified absolute conservation of Ser/Thr125 (highlighted in red) as well as those in the catalytic triad His‐Asp‐Cys (highlighted in yellow), among 2As in all representative enteroviral species obtained from GenBank (Figure 6B, Supporting Information: Figure 6A), showing the importance of the Ser/Thr125 sites in these proteins. These data suggest that phosphorylation also occurs in other enteroviral 2As in residue Thr125. Indeed, the His pull‐down assay identified the predicted phosphorylation at Thr residue(s), but not Ser residue(s), when cells were transfected with His‐Asp‐Cys mutation plasmids of CVB3 2A^pro^ (B3‐2A‐mut) (Figure 6C, Supporting Information: Figure 6B,C). The phosphorylation signal was absent in cells and cell‐free systems with plasmids expressing T125A site‐directed mutation (derived from B3‐2A‐mut), suggesting that single phosphorylation of B3‐2A‐wt occurred at Thr125 (Figure 6D).

Furthermore, we confirmed that Thr125 phosphorylation played the same role in preserving the proteolytic activity of CVB3 2A^pro^ as that in EV‐A71 2A^pro^. As Figure 6E indicates, EGF treatment increased eIF4GI cleavage by B3‐2A‐wt but not phospho‐deficient B3‐2A‐T125A (Figure 6E). The T125E mutants also failed to cleave eIF4GI regardless of the presence or absence of U0126 (Figure 6F). In contrast, cells transfected with the genomic RNA transcript of wt CVB3 Woodruff and its T125A mutated virus provided further evidence that Thr125 phosphorylation of CVB3 2A^pro^ was indispensable for viral propagation, which was analogous to Ser125 phosphorylation of EV‐A71 2A^pro^ (Figure 6G,H, Supporting Information: Figure 6D). Moreover, functional assays demonstrated that the cleavage activity of 2As from five different enteroviral species (EV‐A71, CVB3, PV, EV‐D68, and HRV) on eIF4GI was stronger than that of their individual Ser/Thr125A 2A^pro^ mutants (Figure 6I).

In brief, the results of the phylogenetic analysis and sequence alignments, as well as those of the functional assays, support that the Ser/Thr125 phosphorylation benefits the trans‐catalytic activity of 2A^pro^ and is highly conserved in *Enterovirus*.

## 4 DISCUSSION

The most common strategy for viral infection is to deprive and/or utilize host cell machinery for viral replication.^10^, ^12^, ^20^, ^22^, ^23^, ^24^, ^34^, ^35^, ^36^, ^37^, ^38^, ^39^ Documented evidence shows that the proteolytic activity of enteroviral 2A^pro^ is required for viral proliferation and pathogenesis, by modulating Cap‐to‐IRES translation,^11^, ^12^, ^14^, ^27^ the innate immune antiviral response,^16^, ^17^, ^18^, ^19^ and other processes such as virus‐induced apoptosis.^20^

In this study, we showed that the conserved Ser/Thr125 of 2A^pro^ in *Enterovirus* could be phosphorylated, thereby increasing the catalytic activity of 2A^pro^ and ultimately promoting viral proliferation and pathogenesis, both in vitro and in vivo. Our finding is supported by the following evidences: (i) Ser/Thr125 residues are conserved (Figure 6A,B) and can be phosphorylated in enteroviral 2A^pro^, as observed in cells and cell‐free systems (Figures 2 and 6C,D; Supporting Information: Figure 2C). (ii) Ser/Thr125 phosphorylation plays a key role in enteroviral 2A^pro^ trans‐cleavage, rather than in cis‐cleavage (Figure 3A–F; Supporting Information: Figure 3A,B), viral multiplication (Figure 3G–J; Supporting Information: Figure 3C), and pathogenesis (Figure 4), based on the results of S/T125A (nonphosphor) mutant assays in vitro and in vivo. (iii) The crystal structure simulation revealed that the phosphate groups at Ser125 of EV‐A71 2A^pro^ created a better conformation, facilitating Cys residue to attack the hydrolysis substrate of the catalytic triad (Figure 5, Supporting Information: Figure 5). Taken together, our work supports a model in which Ser125 phosphorylation engages 2A^pro^ via a stable conformation to promote enteroviral 2A trans‐cleavage activity, viral replication, and pathogenesis, based on biological, evolutionary, and structural evidence.

Our previous studies demonstrated that ERK signaling positively regulates enterovirus 2A^pro^ proteolytic function, which is required for enteroviral IRES activity.^27^, ^28^ Here, we demonstrated that Ser/Thr125 at 2A^pro^ of EV‐A71 and CVB3 is the only site that is phosphorylated by active ERKs in cells and cell‐free lysates (Figures 2B,D,G,H and 6D–F). Conversely, when ERK signaling was blocked, Ser/Thr125 phosphorylation was attenuated, thereby decreasing 2A^pro^ catalytic activity (Figures 2B and 6F). Thus, 2A^pro^ phosphorylation at Ser/Thr125 demonstrates that “hijacking” of the host ERK signal is an important adaptive strategy for enteroviral survival. This discovery expands our current understanding of the requirement of ERK functions for enteroviral propagation and pathogenesis, which have been recognized before.^20^, ^21^, ^23^, ^24^, ^27^, ^28^ Since enteroviral 2As are homologous to the trypsin‐like family of serine proteases bearing a His‐Asp‐Ser/Cys center,^33^ we hypothesized that the Ser/Thr residues close to the catalytic Ser/Cys residues at the carboxyl terminus play important roles in these proteases, which are ubiquitous in eukaryotes, prokaryotes, and viruses. Here, we first demonstrated the important action of Ser/Thr residues due to phosphorylation in EVs of *Picornaviridae*. Therefore, our study is in line with Bazan's prediction and suggests that Ser/Thr125 phosphorylation represents a universal regulation of these enzymes from viruses to eukaryotes. Although the leader proteinase (L^pro^) of aphthoviruses in *Picornaviridae* (such as foot‐and‐mouth disease viruses) hydrolyzes eIF4GI to shut down host cellular cap‐dependent translation, similar to those induced by enteroviral 2A protease, the protease is completely different in amino acid sequence alignment, also at position S125 of 2A protease. Therefore, 2A^pro^ with Ser125 phosphorylation sites only exist in EVs of the *Picornaviridae*.

Phosphorylation and dephosphorylation are reversible processes,^30^, ^40^, ^41^, ^42^ and host phosphorylation events contribute to viral life cycles.^20^, ^21^, ^23^, ^24^, ^25^, ^26^, ^27^, ^28^, ^34^, ^35^, ^36^, ^37^, ^38^, ^43^, ^44^, ^45^, ^46^, ^47^ We demonstrated that Ser/Thr125 phosphorylation is required for enteroviral 2A^pro^ to execute its biological functions. Conversely, S125A nonsphosphorylation impaired viral proliferation, thereby reducing their virulence (Figures 3, 4, and 6). Additionally, sera obtained from mice infected with S125A‐rescued EV‐A71 showed neutralizing protection against cells infected with wt viruses (data not shown). Weakened viruses containing a S/T125A mutation are a potential strategy to develop attenuated EV vaccines. Notably, phosphorylation‐mimic mutations (such as S125D) caused deleterious viral proteolytic activity and viral production (Figures 3C–F and 6F), consistent with the findings of crystal structure simulation analysis that the function of S125D may not be able to completely substitute that of the phosphate group (Figure 5). In the case of EV‐A71 2A^pro^, the hydrogen‐bonded network that formed from the phosphate groups of Ser125 phosphorylation maintained the structural conformation required for proteolytic activity, rather than simply creating a negatively charged milieu. This detrimental phenomenon of phosphorylation‐that mimicks mutations is also found in viral p33 of cucumber necrosis virus (CNV)^47^ and the catalytic subunit of cellular protein kinase A.^41^, ^48^ This dogma also applies to other cellular kinases; for example, when the critical S/T residues of p70S6K are mutated to D/E, the protein loses its catalytic function.^49^ Therefore, we provided new evidence that critical Ser/Thr125 residues of enteroviral 2A^pro^ mutated to D/E have deleterious effects on protein function and viral survival.

Due to the low expression of 2A^pro^, we were unable to detect Ser125 phosphorylation using mass spectrometry, indicating that this site was not stoichiometrically phosphorylated. Consistent with this, Ser/Thr125 of enteroviral 2A does not match the phosphorylation motif of ERK1/2. Notably, WB assays for phosphorylated antibodies, ERK activation inhibition assays, and sequence alignment indicate that this site is phosphorylated by ERK and is evolutionarily conserved (Figures 2 and 6). Viral proteins are phosphorylated either at canonical or noncanonical sites by cellular kinases, such as HIV p6 by ERK2^35^ and HSV‐1 VP11/12 by Lck^37^ at canonical sites, rotavirus NSP5 by casein kinase 1^36^ and dengue virus (DENV) NS5 by protein kinase A^38^ at noncanonical sites. Our present study identified Ser/Thr125 as a noncanonical phosphorylation site, where low phosphorylation could be a consequence of selection pressure.

In brief, our biological, structural, and evolutionary profiling data showed that Ser/Thr125 phosphorylation mediated by ERKs, which stabilize a specific conformation, cannot be replaced by D/E acidic residues. This phosphorylation is required for the enteroviral 2A^pro^ function and viral survival. To the best of our knowledge, this is the first study to provide evidence that 2A^pro^ phosphorylation is an important adaptation strategy for enteroviral survival and pathogenesis. Additionally, weakened viruses containing a S/T125A‐mutation are a potential strategy to develop attenuated enterovirus vaccines.

## AUTHOR CONTRIBUTIONS

Yuya Wang contributed to executing experiments. Yuya Wang, Wenjia Zou, and Yihong Peng performed data analysis, interpretation, and manuscript preparation. Sanyuan Wang and Peng Zhang were involved in animal experiments, Bangtao Chen was involved in partial cell experiments, Yihong Peng, Zhijun Luo, and Zhaohua Zhong shared the conceptualization, experimental design, and Yan Niu and Ping Xu simulated the structure of the 2A^pro^ phosphorylation. Sanyuan Wang, Changfa Fan, Rui Xiong, and Yuya Wang provided the animal model and relative experiment design.

## CONFLICT OF INTEREST

The authors declare no conflict of interest.

## Supporting information

Supplementary information.

## Data Availability

The data that support the findings of this study are available upon reasonable request.

## References

[1] Zell R , Delwart E , Gorbalenya AE , et al. ICTV virus taxonomy profile: picornaviridae. J Gen Virol. 2017;98(10):2421‐2422. 28884666 10.1099/jgv.0.000911 PMC5725991

[2] McIntyre CL , Knowles NJ , Simmonds P . Proposals for the classification of human rhinovirus species A, B and C into genotypically assigned types. J Gen Virol. 2013;94(Pt 8):1791‐1806. 23677786 10.1099/vir.0.053686-0 PMC3749525

[3] Fischer TK , Simmonds P , Harvala H . The importance of enterovirus surveillance in a post‐polio world. Lancet Infect Dis. 2022;22(1):e35‐e40. 34265258 10.1016/S1473-3099(20)30852-5

[4] Hu Y , Musharrafieh R , Zheng M , Wang J . Enterovirus D68 antivirals: past, present, and future. ACS Infect Dis. 2020;6(7):1572‐1586. 32352280 10.1021/acsinfecdis.0c00120 PMC8055446

[5] Kim KS , Hufnagel G , Chapman NM , Tracy S . The group B coxsackieviruses and myocarditis. Rev Med Virol. 2001;11(6):355‐368. 11746998 10.1002/rmv.326

[6] Precechtelova J , Borsanyiova M , Sarmirova S , Bopegamage S . Type I diabetes mellitus: genetic factors and presumptive enteroviral etiology or protection. J Pathog. 2014;2014:738512. 25574400 10.1155/2014/738512 PMC4276674

[7] Baggen J , Thibaut HJ , Strating JRPM , van Kuppeveld FJM . The life cycle of non‐polio enteroviruses and how to target it. Nat Rev Microbiol. 2018;16(6):368‐381. 29626210 10.1038/s41579-018-0005-4

[8] Kitamura N , Semler BL , Rothberg PG , et al. Primary structure, gene organization and polypeptide expression of poliovirus RNA. Nature. 1981;291(5816):547‐553. 6264310 10.1038/291547a0

[9] Lulla V , Dinan AM , Hosmillo M , et al. An upstream protein‐coding region in enteroviruses modulates virus infection in gut epithelial cells. Nat Microbiol. 2019;4(2):280‐292. 30478287 10.1038/s41564-018-0297-1 PMC6443042

[10] Borman AM , Le Mercier P , Girard M , Kean KM . Comparison of picornaviral IRES‐driven internal initiation of translation in cultured cells of different origins. Nucleic Acids Res. 1997;25(5):925‐932. 9023100 10.1093/nar/25.5.925 PMC146526

[11] Kräusslich HG , Nicklin MJ , Toyoda H , Etchison D , Wimmer E . Poliovirus proteinase 2A induces cleavage of eucaryotic initiation factor 4F polypeptide p220. J Virol. 1987;61(9):2711‐2718. 3039165 10.1128/jvi.61.9.2711-2718.1987 PMC255777

[12] Lee K‐M , Chen C‐J , Shih S‐R . Regulation mechanisms of viral IRES‐driven translation. Trends Microbiol. 2017;25(7):546‐561. 28242053 10.1016/j.tim.2017.01.010

[13] Yu SF , Lloyd RE . Identification of essential amino acid residues in the functional activity of poliovirus 2A protease. Virology. 1991;182(2):615‐625. 1850921 10.1016/0042-6822(91)90602-8

[14] Castelló A , Alvarez E , Carrasco L . The multifaceted poliovirus 2A protease: regulation of gene expression by picornavirus proteases. J Biomed Biotechnol. 2011;2011:369648. 21541224 10.1155/2011/369648 PMC3085340

[15] Yang X , Cheng A , Wang M , et al. Structures and corresponding functions of five types of picornaviral 2A proteins. Front Microbiol. 2017;8:1373. 28785248 10.3389/fmicb.2017.01373 PMC5519566

[16] Kuo R‐L , Chen C‐J , Wang RYL , et al. Role of enteroviral RNA‐dependent RNA polymerase in regulation of MDA5‐mediated beta interferon activation. J Virol. 2019;93(10):e00132‐19. 30814289 10.1128/JVI.00132-19 PMC6498057

[17] Wang B , Xi X , Lei X , et al. Enterovirus 71 protease 2A^pro^ targets MAVS to inhibit anti‐viral type I interferon responses. PLoS Pathog. 2013;9(3):e1003231. 23555247 10.1371/journal.ppat.1003231 PMC3605153

[18] Lu J , Yi L , Zhao J , et al. Enterovirus 71 disrupts interferon signaling by reducing the level of interferon receptor 1. J Virol. 2012;86(7):3767‐3776. 22258259 10.1128/JVI.06687-11 PMC3302529

[19] Chen B , Wang Y , Pei X , Wang S , Zhang H , Peng Y . Cellular caspase‐3 contributes to EV‐A71 2A^pro^‐mediated down‐regulation of IFNAR1 at the translation level. Virol Sin. 2020;35(1):64‐72. 31512106 10.1007/s12250-019-00151-y PMC7035401

[20] Jensen KJ , Garmaroudi FS , Zhang J , et al. An ERK‐p38 subnetwork coordinates host cell apoptosis and necrosis during coxsackievirus B3 infection. Cell Host Microbe. 2013;13(1):67‐76. 23332156 10.1016/j.chom.2012.11.009 PMC3553504

[21] Luo H , Yanagawa B , Zhang J , et al. Coxsackievirus B3 replication is reduced by inhibition of the extracellular signal‐regulated kinase (ERK) signaling pathway. J Virol. 2002;76(7):3365‐3373. 11884562 10.1128/JVI.76.7.3365-3373.2002 PMC136021

[22] Pleschka S . RNA viruses and the mitogenic Raf/MEK/ERK signal transduction cascade. Biol Chem. 2008;389(10):1273‐1282. 18713014 10.1515/BC.2008.145

[23] Wang B , Zhang H , Zhu M , Luo Z , Peng Y . MEK1‐ERKs signal cascade is required for the replication of Enterovirus 71 (EV71). Antiviral Res. 2012;93(1):110‐117. 22101247 10.1016/j.antiviral.2011.11.001

[24] Brown MC , Dobrikov MI , Gromeier M . Mitogen‐activated protein kinase‐interacting kinase regulates mTOR/AKT signaling and controls the serine/arginine‐rich protein kinase‐responsive type 1 internal ribosome entry site‐mediated translation and viral oncolysis. J Virol. 2014;88(22):13149‐13160. 25187540 10.1128/JVI.01884-14 PMC4249080

[25] Jheng J‐R , Wang S‐C , Jheng C‐R , Horng J‐T . Enterovirus 71 induces dsRNA/PKR‐dependent cytoplasmic redistribution of GRP78/BiP to promote viral replication. Emerg Microbes Infect. 2016;5:1‐12. 10.1038/emi.2016.20 PMC4820672 27004760

[26] Wang C , Sun M , Yuan X , et al. Enterovirus 71 suppresses interferon responses by blocking Janus kinase (JAK)/signal transducer and activator of transcription (STAT) signaling through inducing karyopherin‐α1 degradation. J Biol Chem. 2017;292(24):10262‐10274. 28455446 10.1074/jbc.M116.745729 PMC5473229

[27] Duan H , Zhu M , Xiong Q , et al. Regulation of enterovirus 2A protease‐associated viral IRES activities by the cell's ERK signaling cascade: implicating ERK as an efficiently antiviral target. Antiviral Res. 2017;143:13‐21. 28351508 10.1016/j.antiviral.2017.03.018

[28] Zhang H , Wang X , Wang Y , et al. Substituted 3‐benzylcoumarins 13 and 14 suppress enterovirus A71 replication by impairing viral 2A^pro^ dependent IRES‐driven translation. Antiviral Res. 2018;160:10‐16. 30315876 10.1016/j.antiviral.2018.10.012

[29] Cargnello M , Roux PP . Activation and function of the MAPKs and their substrates, the MAPK‐activated protein kinases. Microbiol Mol Biol Rev. 2011;75(1):50‐83. 21372320 10.1128/MMBR.00031-10 PMC3063353

[30] Yoon S , Seger R . The extracellular signal‐regulated kinase: multiple substrates regulate diverse cellular functions. Growth Factors. 2006;24(1):21‐44. 16393692 10.1080/02699050500284218

[31] Imura A , Sudaka Y , Takashino A , et al. Development of an enterovirus 71 vaccine efficacy test using human scavenger receptor B2 transgenic mice. J Virol. 2020;94(6):e01921‐19. 31896594 10.1128/JVI.01921-19 PMC7158731

[32] Cai Q , Yameen M , Liu W , et al. Conformational plasticity of the 2A proteinase from enterovirus 71. J Virol. 2013;87(13):7348‐7356. 23616646 10.1128/JVI.03541-12 PMC3700279

[33] Bazan JF , Fletterick RJ . Viral cysteine proteases are homologous to the trypsin‐like family of serine proteases: structural and functional implications. Proc Natl Acad Sci USA. 1988;85(21):7872‐7876. 3186696 10.1073/pnas.85.21.7872 PMC282299

[34] Pleschka S , Wolff T , Ehrhardt C , et al. Influenza virus propagation is impaired by inhibition of the Raf/MEK/ERK signalling cascade. Nature Cell Biol. 2001;3(3):301‐305. 11231581 10.1038/35060098

[35] Hemonnot B , Cartier C , Gay B , et al. The host cell MAP kinase ERK‐2 regulates viral assembly and release by phosphorylating the p6 protein of HIV‐1. J Biol Chem. 2004;279(31):32426‐32434. 15155723 10.1074/jbc.M313137200

[36] Eichwald C , Jacob G , Muszynski B , Allende JE , Burrone OR . Uncoupling substrate and activation functions of rotavirus NSP5: phosphorylation of Ser‐67 by casein kinase 1 is essential for hyperphosphorylation. Proc Natl Acad Sci USA. 2004;101(46):16304‐16309. 15520389 10.1073/pnas.0406691101 PMC528968

[37] Zahariadis G , Wagner MJ , Doepker RC , et al. Cell‐type‐specific tyrosine phosphorylation of the herpes simplex virus tegument protein VP11/12 encoded by gene UL46. J Virol. 2008;82(13):6098‐6108. 18417566 10.1128/JVI.02121-07 PMC2447066

[38] Bhattacharya D , Mayuri I , Best SM , Perera R , Kuhn RJ , Striker R . Protein kinase G phosphorylates mosquito‐borne flavivirus NS5. J Virol. 2009;83(18):9195‐9205. 19587048 10.1128/JVI.00271-09 PMC2738234

[39] Li Z , Yang X , Zhao Z , Liu X , Zhang W . Host restriction factor A3G inhibits the replication of enterovirus D68 by competitively binding the 5' untranslated region with PCBP1. J Virol. 2022;96(2):e0170821. 34730395 10.1128/JVI.01708-21 PMC8791300

[40] Manning G , Whyte DB , Martinez R , Hunter T , Sudarsanam S . The protein kinase complement of the human genome. Science. 2002;298(5600):1912‐1934. 12471243 10.1126/science.1075762

[41] Steichen JM , Iyer GH , Li S , et al. Global consequences of activation loop phosphorylation on protein kinase A. J Biol Chem. 2010;285(6):3825‐3832. 19965870 10.1074/jbc.M109.061820 PMC2823524

[42] Whitmarsh AJ , Davis RJ . Multisite phosphorylation by MAPK. Science. 2016;354(6309):179‐180. 27738159 10.1126/science.aai9381

[43] He Z , He YS , Kim Y , et al. The human cytomegalovirus UL97 protein is a protein kinase that autophosphorylates on serines and threonines. J Virol. 1997;71(1):405‐411. 8985364 10.1128/jvi.71.1.405-411.1997 PMC191065

[44] Modrof J , Möritz C , Kolesnikova L , et al. Phosphorylation of Marburg virus VP30 at serines 40 and 42 is critical for its interaction with NP inclusions. Virology. 2001;287(1):171‐182. 11504552 10.1006/viro.2001.1027

[45] Newsome TP , Weisswange I , Frischknecht F , Way M . Abl collaborates with Src family kinases to stimulate actin‐based motility of vaccinia virus. Cell Microbiol. 2006;8(2):233‐241. 16441434 10.1111/j.1462-5822.2005.00613.x

[46] Leong SY , Ong BKT , Chu JJH . The role of Misshapen NCK‐related kinase (MINK), a novel Ste20 family kinase, in the IRES‐mediated protein translation of human enterovirus 71. PLoS Pathog. 2015;11(3):e1004686. 25747578 10.1371/journal.ppat.1004686 PMC4352056

[47] Stork J , Panaviene Z , Nagy PD . Inhibition of in vitro RNA binding and replicase activity by phosphorylation of the p33 replication protein of cucumber necrosis tombusvirus. Virology. 2005;343(1):79‐92. 16154612 10.1016/j.virol.2005.08.005

[48] Yonemoto W , McGlone ML , Grant B , Taylor SS . Autophosphorylation of the catalytic subunit of cAMP‐dependent protein kinase in *Escherichia coli* . Protein Eng Des Sel. 1997;10(8):915‐925. 10.1093/protein/10.8.915 9415441

[49] McClendon CL , Kornev AP , Gilson MK , Taylor SS . Dynamic architecture of a protein kinase. Proc Natl Acad Sci USA. 2014;111(43):E4623‐E4631. 25319261 10.1073/pnas.1418402111 PMC4217441

