# Supplementary material for: Phosphorylation of enteroviral 2Apro at Ser/Thr125 benefits its proteolytic activity and viral pathogenesis
Source: J Med Virol. 2022 Dec 22;95(1):e28400. doi: 10.1002/jmv.28400 (PMC10107306; doi:10.1002/jmv.28400)
Supplement: Supplementary file 1 — Supplementary information. [file JMV-95-0-s001.docx]

Supplementary Materials

[Supplementary Table 1 The full and abbreviated names of all plasmids. 2](#_Toc119700696)

[Supplementary Table 2 Primers of plasmids used in this study. 4](#_Toc119700697)

[Supplementary Table 3 siRNA used in this study 6](#_Toc119700698)

[Supplementary Table 4 Primers of infectious clone used in this study. 8](#_Toc119700699)

[Supplementary Table 5 Clinical signs record chart. 9](#_Toc119700700)

[Supplementary Figure 1 24](#_Toc119700701)

[Supplementary Figure 2 25](#_Toc119700702)

[Supplementary Figure 3 27](#_Toc119700703)

[Supplementary Figure 4 29](#_Toc119700704)

[Supplementary Figure 5 30](#_Toc119700705)

[Supplementary Figure 6 31](#_Toc119700706)

# Supplementary Table 1 The full and abbreviated names of all plasmids.

| **Plasmid Abbreviation** | **Plasmid Full Name** |
| --- | --- |
| A71-2A-wt | pcDNA3.1 EGFP-EV-A71 2A^pro^-His |
| A71-2A-mut | pcDNA3.1-EGFP- EV-A71 2A^mut^-His |
| A71-2A-S125A | pcDNA3.1-EGFP-A71 2A^pro-S125A^-His |
| A71-2A-mut-S7A | pcDNA3.1-EGFP- EV-A71 2A^mut-S7A^-His |
| A71-2A-mut-S36A | pcDNA3.1-EGFP- EV-A71 2A^mut-S36A^-His |
| A71-2A-mut-S37A | pcDNA3.1-EGFP- EV-A71 2A^mut-S37A^-His |
| A71-2A-mut-S43A | pcDNA3.1-EGFP- EV-A71 2A^mut-S43A^-His |
| A71-2A-mut-S44A | pcDNA3.1-EGFP- EV-A71 2A^mut-S44A^-His |
| A71-2A-mut-S67A | pcDNA3.1-EGFP- EV-A71 2A^mut-S67A^-His |
| A71-2A-mut-S75A | pcDNA3.1-EGFP- EV-A71 2A^mut-S75A^-His |
| A71-2A-mut-S77A | pcDNA3.1-EGFP- EV-A71 2A^mut-S77A^-His |
| A71-2A-mut-S80A | pcDNA3.1-EGFP- EV-A71 2A^mut-S80A^-His |
| A71-2A-mut-S87A | pcDNA3.1-EGFP- EV-A71 2A^mut-S87A^-His |
| A71-2A-mut-96A | pcDNA3.1-EGFP- EV-A71 2A^mut-S96A^-His |
| A71-2A-mut-S105A | pcDNA3.1-EGFP- EV-A71 2A^mut-S105A^-His |
| A71-2A-mut-S125A | pcDNA3.1-EGFP- EV-A71 2A^mut-S125A^-His |
| B3-2A-wt | pcDNA3.1 EGFP-CVB3 2A^pro^-His |
| B3-2A-mut | pcDNA3.1-EGFP- CVB3 2A^mut^-His |
| B3-2A-mut-T125A | pcDNA3.1-EGFP- CVB3 2A^mut-T125A^-His |
| B3-2A-T125A  B3-2A-T125E | pcDNA3.1-EGFP- CVB3 2A^T125A^-His  pcDNA3.1-EGFP- CVB3 2A^T125E^-His |
| PV wt | pcDNA3.1-EGFP-PV 2A^pro^-His |
| PV T-to-A | pcDNA3.1-EGFP-PV 2A^T-to-A^ -His |
| EV-D68 wt | pcDNA3.1-EGFP- EV-D68 2A^pro^-His |
| EV-D68 T-to-A | pcDNA3.1-EGFP- EV-D68 2A^T-to-A^ -His |
| HRV wt | pcDNA3.1-EGFP- RV 2A^pro^ -His |
| HRV T-to-A | pcDNA3.1-EGFP- RV 2A^T-to-A^ -His |
| P1-2A-wt | pET21a-EV-A71 P1-2A^pro^ |
| P1-2A-S125A | pET21a-EV-A71 P1-2A^S125A^ |
| P1-2A-S125D | pET21a-EV-A71 P1-2A^S125D^ |

# Supplementary Table 2 Primers of plasmids used in this study.

| **Primers name** |  | **Sequences(5’-3’)** |
| --- | --- | --- |
| A71-2A-wt/  A71-2A-mut | sense | CATCATCACCATCACCATTCTAGAGGGCCCGTTTAAACC |
|  | antisense | ATGATGGTGATGGTGATGCTGTTCCATAGCTTCTTCATCCAAC |
| B3-2A-wt/  B3-2A-mut | sense | CATCATCACCATCACCATTAATCTAGAGGGCCCGTTTAAAC |
|  | antisense | ATGGTGATGGTGATGATGCTGTTCCATTGCATCATCTTC |
| A71-2A-mut-S7A | sense | AAATTTGGACAACAGGCCGGGGCCATTTATGTGGG |
|  | antisense | \| CTGTTGTCCAAATTTCCCAAG \| \| --- \| |
| A71-2A-mut-S36A | sense | \| CTTGTTTGGGAAGACGCTTCTCGCGAATTGCTCGT \| \| --- \| |
|  | antisense | CAAATCTTGTTTGGGAAGAC |
| A71-2A-mut-S37A | sense | CTTGTTTGGGAAGACAGTGCTCGCGAATTGCTCG |
|  | antisense | GTCTTCCCAAACAAGATTTG |
| A71-2A-mut-S43A | sense | CGCGAATTGCTCGTAGCATCCACCACCGCCCAAGG |
|  | antisense | TACGAGCAATTCGCGAGAAC |
| A71-2A-mut-S44A | sense | GAATTGCTCGTATCAGCCACCACCGCCCAAGGTTG |
|  | antisense | TGATACGAGCAATTCGCGAG |
| A71-2A-mut-S67A | sense | GTGTACTACTGTAACGCGAGGAGAAAACACTACCC |
|  | antisense | GTTACAGTAGTACACCCCTG |
| A71-2A-mut-S75A | sense | AAACACTACCCAGTCGCCTTTTCAAAACCCAGCC |
|  | antisense | GACTGGGTAGTGTTTTCTCC |
| A71-2A-mut-S77A | sense | ACTACCCAGTCAGCTTTGCAAAACCCAGCCTGATC |
|  | antisense | AGCTGACTGGGTAGTGTTTTC |
| A71-2A-mut-S80A | sense | AGCTTTTCAAAACCCGCCCTGATCTATGTAGAGGC |
|  | antisense | GGGTTTTGAAAAGCTGACTG |
| A71-2A-mut-S87A | sense | ATCTATGTAGAGGCTGCCGAGTATTACCCAGCCAG |
|  | antisense | AGCCTCTACATAGATCAGGC |
| A71-2A-mut-S96A | sense | CCAGCCAGGTACCAAGCACATCTCATGCTCGCACA |
|  | antisense | TTGGTACCTGGCTGGGTAATACT |
| A71-2A-mut-S105A | sense | CTCGCACCGGGTCACGCAGAGCCTGGCGATGCCG |
|  | antisense | GTGACCCGGTGCGAGCATGA |
| A71-2A-mut-S125A/A71-2A-wt-S125A | sense | GTCGTCGGCATAGTGGCTACTGGTGGCAATGGGCT |
|  | antisense | CACTATGCCGACGACGCCAT |
| B3-2A-mut-S125A/B3-2A-wt-S125A | sense | ATTGTGGCCATGGGGGGTGAAGGCGTGGTCGGCTT |
|  | antisense | CCCCATGGCCACAATGCCAATGACACCATGCTCACA |
| pVector-His | sense | CATCATCACCATCACCATTCTAGAGGGCCCGTTTAAACCC |
|  | antisense | ATGGTGATGGTGATGATGCTTCGATCCGCCACCGCCAGA |
| A71-2A-S125D | sense | GTCGGCATAGTGGATACTGGTGGCAATGGGCTCGTT |
|  | antisense | ATCCACTATGCCGACGACGCC |
| B3-2A-T125E | sense | CATTG TGGAA ATGGGGGGTGAAGGCGTGGTCGGCT |
|  | antisense | CCCATTTCCACAATGCCAATGAC |
| PV wt | sense | GGCGGATCGAAGCTTGGATTCGGACACCAAAACAAAG |
|  | antisense | GTGATGGTGATGATGTTGTTCCATGGCTTCTTCTTCG |
| PV T125A | sense | AGGGATCATTGCTGCTGGTGGCGAAGGGTTGGTTG |
|  | antisense | GCAGCAATGATCCCTATCACC |
| EV-D68 wt | sense | GGCGG ATCGA AGCTTGGTCCAGGTT TTGGGGGAGT |
|  | antisense | GTGATGGTGATGATGTTGTTCCATAACATCAGTATCTA |
| EV-D68 T125A | sense | TGGCCTCCTTGCAGCAGGAGGGGGTGGGATTGTAG |
|  | antisense | GCTGCAAGGAGGCCAATCAC |
| HRV wt | sense | GGCGG ATCGA AGCTT GGGCCCAGTG ATCTATATGT G |
|  | antisense | GTGATGGTGATGATGTTGTTCCTCAGCACAGTGAAAT |
| HRV T125A | sense | TGGCATAATCGCAGCAGGTGGTGAAGGTCATGTAGCA |
|  | antisense | GCTGCGATTATGCCAATGACAC |
| pET21a P1-2A-wt | sense | GGATTCGCGATGGGCTCGCAAGTGTCTAC |
|  | antisense | CTCGAGCGGCTGTTCCATAGCTTCTTCATC |
| pET21a P1-2A-S125A | sense | GTCGTCGGCATAGTGGCTACTGGTGGCAATGGGCT |
|  | antisense | CACTATGCCGACGACGCCAT |
| pIRES P1-2A-wt | sense | GAAGCTATGGAACAGAGGTTACACACACCTCAACC |
|  | antisense | CTGTTCCATAGCTTCTTCATC |
| pIRES P1-2A-S125A | sense | GTCGTCGGCATAGTGGCTACTGGTGGCAATGGGCT |
| pIRES P1 | antisense  sense  antisense | CACTATGCCGACGACGCCAT  AAGAGTGGTGATCGCTGTGC  GCGATCACCACTCTT AGGTTACACACACCTCAACC |

| **gene** |  | **Sequences(5’-3’)** |
| --- | --- | --- |
| ERK1(Homo) | sense | CCCUGACCCGUCUAAUAUAdTdT |
|  | antisense | UAUAUUAGACGGGUCAGGGdTdT |
| ERK2(Homo) | sense | CAUGGUAGUCACUAACAUAdTdT |
|  | antisense | UAUGUUAGUGACUACCAUGdTdT |
| msiERK1(Homo) | sense | CCCUGACCCGUCUAAAUUAdTdT |
|  | antisense | UAAUUUAGACGGGUCAGGGdTdT |
| msiERK2(Homo) | sense | CAUGGUAGUCACUAAACUAdTdT |
|  | antisense | UAGUUUAGUGACUACCAUGdTdT |

# Supplementary Table 3 siRNA used in this study

**Supplementary Table 3 Primers of infectious clone used in this study.**

| gene |  | Sequences(5’-3’) |
| --- | --- | --- |
| -T7-rbz-5’UTR- | sense | TAATACGACTCACTATAGG |
|  | antisense | GTTTGGCTGCGTTAAGGGTC |
| -P1- | sense | TTAACGCAGCCAAACATGGGCTCGCAAGTGTCTAC |
|  | antisense | AAGAGTGGTGATCGCTGTGC |
| -P2-P3-3’UTR- | sense | GCGATCACCACTCTTGGGAAATTTGGCCAACAGTCT |
|  | antisense | TAG AAT ACT TTG AGT ATT CTA TAG TGT CA |
| -pBR322- | sense | TGCATAGCTTGAGTATTCTATCATCGATAAGCTTTAATGCGGTAG |
|  | antisense | CTATAGTGAGTCGTATTATAAGCTGTCAAACATGAGAATTC |
| -B3- | sense | CGGTATCCCGGGTTCTTAAAACAGCCTGTGGGTTGAT |
|  | antisense | TTACCCCTACTGCACCGTTGTCTAGTTCGGTTAGTA |
|  | fragment | GTGCAGTAGGGGTAAATTCTCCGCATTCGGTGCGGAAAAAAAAAAAAAAAAAAAAAAAAAAAAAAAAAAAAAATTGCATAGCTT GAGTATTCTATCATC |
| -B3-pBR322- | sense | CATAGCTTGAGTATTCTATCATC |
|  | antisense | GAACCCGGGATACCGGGTTTTCGGCCTTTCGGCCT |
| -2A-S125A-/-2A-S125D- | sense | GGGAAATTTGGACAACAGTCTG |
|  | antisense | CTGTTCCATAGCTTCTTCATC |
| -A71- | sense | ATGAAGAAGCTATGGAACAG |
|  | antisense | TTGTCCAAATTTCCCAAGAGTG |
| -2A-T125A -/-2A-T125D- | sense | GGCGCATTTGGACAACAATC |
|  | antisense | CTGTTCCATTGCATCATCTTC |
| -B3- | sense | GAAGATGATGCAATGGAACAG |
|  | antisense | GATTGTTGTCCAAATGCGCC |
| A71-seq | sense | CACTCCAAGCTGCTGAAATTG |
|  | antisense | CCGCCCTACTGAAGAAACTATC |
| B3-seq | sense | CACTCACTGCTGCTGAGACAG |
|  | antisense | CTGACCTACATAGGAAGTTCTC |
| S125A EV-A71 | sense | GTCGTCGGCATAGTGGCTACTGGTGGCAATGGGCT |
|  | antisense | CACTATGCCGACGACGCCAT |
| T125A CVB3 | sense | ATTGTGGCCATGGGGGGTGAAGGCGTGGTCGGCTT |
|  | antisense | CCCCATGGCCACAATGCCAATGACACCATGCTCACA |

# Supplementary Table 4 Primers of infectious clone used in this study.

| **gene** |  | **Sequences(5’-3’)** |
| --- | --- | --- |
| -T7-rbz-5’UTR- | sense | TAATACGACTCACTATAGG |
|  | antisense | GTTTGGCTGCGTTAAGGGTC |
| -P1- | sense | TTAACGCAGCCAAACATGGGCTCGCAAGTGTCTAC |
|  | antisense | AAGAGTGGTGATCGCTGTGC |
| -P2-P3-3’UTR- | sense | GCGATCACCACTCTTGGGAAATTTGGCCAACAGTCT |
|  | antisense | TAG AAT ACT TTG AGT ATT CTA TAG TGT CA |
| -pBR322- | sense | TGCATAGCTTGAGTATTCTATCATCGATAAGCTTTAATGCGGTAG |
|  | antisense | CTATAGTGAGTCGTATTATAAGCTGTCAAACATGAGAATTC |
| -B3- | sense | CGGTATCCCGGGTTCTTAAAACAGCCTGTGGGTTGAT |
|  | antisense | TTACCCCTACTGCACCGTTGTCTAGTTCGGTTAGTA |
|  | fragment | GTGCAGTAGGGGTAAATTCTCCGCATTCGGTGCGGAAAAAAAAAAAAAAAAAAAAAAAAAAAAAAAAAAAAAATTGCATAGCTT GAGTATTCTATCATC |
| -B3-pBR322- | sense | CATAGCTTGAGTATTCTATCATC |
|  | antisense | GAACCCGGGATACCGGGTTTTCGGCCTTTCGGCCT |
| -2A-S125A-/-2A-S125D- | sense | GGGAAATTTGGACAACAGTCTG |
|  | antisense | CTGTTCCATAGCTTCTTCATC |
| -A71- | sense | ATGAAGAAGCTATGGAACAG |
|  | antisense | TTGTCCAAATTTCCCAAGAGTG |
| -2A-T125A -/-2A-T125D- | sense | GGCGCATTTGGACAACAATC |
|  | antisense | CTGTTCCATTGCATCATCTTC |
| -B3- | sense | GAAGATGATGCAATGGAACAG |
|  | antisense | GATTGTTGTCCAAATGCGCC |
| A71-seq | sense | CACTCCAAGCTGCTGAAATTG |
|  | antisense | CCGCCCTACTGAAGAAACTATC |
| B3-seq | sense | CACTCACTGCTGCTGAGACAG |
|  | antisense | CTGACCTACATAGGAAGTTCTC |
| S125A EV-A71 | sense | GTCGTCGGCATAGTGGCTACTGGTGGCAATGGGCT |
|  | antisense | CACTATGCCGACGACGCCAT |
| T125A CVB3 | sense | ATTGTGGCCATGGGGGGTGAAGGCGTGGTCGGCTT |
|  | antisense | CCCCATGGCCACAATGCCAATGACACCATGCTCACA |

# Supplementary Table 5 Clinical signs record chart.

| Clinical signs record chart | | | | | | | | | |
| --- | --- | --- | --- | --- | --- | --- | --- | --- | --- |
| 1 day’s post infection | | | | | | | | | |
|  | | | | | | | | | |
|  | **ruffled fur**  **（0-5）** | **Hunchbacked** | **slow in action** | **Limb weakness/ Limb trembling** | **Limb paralysis** | **Weight loss** | **Moribund and death** | **sum** |  |
|  |  | **（0-5）** | **（0-5）** | **（0-5）** | **（0-5）** | **（0-5）** | **35** | **（0-35）** |  |
| **WT-1** | 0 | 0 | 0 | 0 | 0 | 5 | 0 | 5 |  |
| **WT-2** | 1 | 1 | 0 | 0 | 0 | 5 | 0 | 7 |  |
| **WT-3** | 0 | 0 | 0 | 0 | 0 | 5 | 0 | 5 |  |
| **WT-4** | 1 | 2 | 0 | 0 | 0 | 5 | 0 | 8 |  |
| **WT-5** | 0 | 0 | 0 | 0 | 0 | 0 | 0 | 0 |  |
| **WT-6** | 2 | 2 | 1 | 0 | 0 | 5 | 0 | 10 |  |
| **WT-7** | 0 | 0 | 0 | 0 | 0 | 5 | 0 | 5 |  |
| **S125A-1** | 2 | 1 | 2 | 0 | 0 | 5 | 0 | 10 |  |
| **S125A-2** | 0 | 0 | 0 | 0 | 0 | 5 | 0 | 5 |  |
| **S125A-3** | 1 | 0 | 0 | 0 | 0 | 5 | 0 | 6 |  |
| **S125A-4** | 0 | 1 | 2 | 0 | 0 | 5 | 0 | 8 |  |
| **S125A-5** | 0 | 0 | 0 | 0 | 0 | 5 | 0 | 5 |  |
| **S125A-6** | 0 | 0 | 0 | 0 | 0 | 5 | 0 | 5 |  |
| **S125A-7** | 1 | 1 | 1 | 0 | 0 | 5 | 0 | 8 |  |
| **Mock-1** | 0 | 0 | 0 | 0 | 0 | 0 | 0 | 0 |  |
| **Mock-2** | 0 | 0 | 0 | 0 | 0 | 0 | 0 | 0 |  |
| **Mock-3** | 0 | 0 | 0 | 0 | 0 | 0 | 0 | 0 |  |
| **Mock-4** | 0 | 0 | 0 | 0 | 0 | 0 | 0 | 0 |  |
| **Mock-5** | 0 | 0 | 0 | 0 | 0 | 0 | 0 | 0 |  |
| **Mock-6** | 0 | 0 | 0 | 0 | 0 | 0 | 0 | 0 |  |
| **Mock-7** | 0 | 0 | 0 | 0 | 0 | 0 | 0 | 0 |  |

“×” means death.

| Clinical signs record chart | | | | | | | | | |
| --- | --- | --- | --- | --- | --- | --- | --- | --- | --- |
| 2 day’s post infection | | | | | | | | | |
|  | | | | | | | | | |
|  | **ruffled fur**  **（0-5）** | **Hunchbacked** | **slow in action** | **Limb weakness/ Limb trembling** | **Limb paralysis** | **Weight loss** | **Moribund and death** | **sum** |  |
|  |  | **（0-5）** | **（0-5）** | **（0-5）** | **（0-5）** | **（0-5）** | **35** | **（0-35）** |  |
| **WT-1** | 0 | 0 | 0 | 0 | 0 | 5 | 0 | 5 |  |
| **WT-2** | 0 | 0 | 0 | 0 | 0 | 5 | 0 | 5 |  |
| **WT-3** | 2 | 0 | 0 | 0 | 0 | 5 | 0 | 7 |  |
| **WT-4** | 0 | 1 | 0 | 0 | 0 | 5 | 0 | 6 |  |
| **WT-5** | 0 | 0 | 0 | 0 | 0 | 0 | 0 | 0 |  |
| **WT-6** | 0 | 0 | 0 | 0 | 0 | 5 | 0 | 5 |  |
| **WT-7** | 2 | 3 | 2 | 0 | 0 | 5 | 0 | 12 |  |
| **S125A-1** | 0 | 0 | 0 | 0 | 0 | 0 | 0 | 0 |  |
| **S125A-2** | 0 | 0 | 0 | 0 | 0 | 0 | 0 | 0 |  |
| **S125A-3** | 0 | 0 | 0 | 0 | 0 | 0 | 0 | 0 |  |
| **S125A-4** | 0 | 0 | 0 | 0 | 0 | 0 | 0 | 0 |  |
| **S125A-5** | 0 | 1 | 2 | 0 | 0 | 0 | 0 | 3 |  |
| **S125A-6** | 0 | 1 | 1 | 0 | 0 | 0 | 0 | 2 |  |
| **S125A-7** | 0 | 1 | 0 | 0 | 0 | 5 | 0 | 6 |  |
| **Mock-1** | 0 | 0 | 0 | 0 | 0 | 0 | 0 | 0 |  |
| **Mock-2** | 0 | 0 | 0 | 0 | 0 | 0 | 0 | 0 |  |
| **Mock-3** | 0 | 0 | 0 | 0 | 0 | 0 | 0 | 0 |  |
| **Mock-4** | 0 | 0 | 0 | 0 | 0 | 0 | 0 | 0 |  |
| **Mock-5** | 0 | 0 | 0 | 0 | 0 | 0 | 0 | 0 |  |
| **Mock-6** | 0 | 0 | 0 | 0 | 0 | 0 | 0 | 0 |  |
| **Mock-7** | 0 | 0 | 0 | 0 | 0 | 0 | 0 | 0 |  |

“×” means death.

| Clinical signs record chart | | | | | | | | |
| --- | --- | --- | --- | --- | --- | --- | --- | --- |
| 3 day’s post infection | | | | | | | | |
|  | | | | | | | | |
|  | **ruffled fur**  **（0-5）** | **Hunchbacked** | **slow in action** | **Limb weakness/ Limb trembling** | **Limb paralysis** | **Weight loss** | **Moribund and death** | **sum** |
|  |  | **（0-5）** | **（0-5）** | **（0-5）** | **（0-5）** | **（0-5）** | **35** | **（0-35）** |
| **WT-1** | 0 | 0 | 0 | 0 | 0 | 5 | 0 | 5 |
| **WT-2** | 1 | 2 | 0 | 0 | 0 | 0 | 0 | 3 |
| **WT-3** | 0 | 0 | 0 | 0 | 0 | 0 | 0 | 0 |
| **WT-4** | 2 | 2 | 0 | 0 | 0 | 0 | 0 | 4 |
| **WT-5** | 0 | 0 | 0 | 0 | 0 | 0 | 0 | 0 |
| **WT-6** | 1 | 0 | 2 | 0 | 0 | 5 | 0 | 8 |
| **WT-7** | 3 | 2 | 2 | 1 | 0 | 0 | 0 | 8 |
| **S125A-1** | 1 | 0 | 0 | 0 | 0 | 0 | 0 | 1 |
| **S125A-2** | 0 | 0 | 0 | 0 | 0 | 0 | 0 | 0 |
| **S125A-3** | 0 | 0 | 0 | 0 | 0 | 0 | 0 | 0 |
| **S125A-4** | 1 | 0 | 0 | 0 | 0 | 0 | 0 | 1 |
| **S125A-5** | 0 | 0 | 0 | 0 | 0 | 0 | 0 | 0 |
| **S125A-6** | 2 | 2 | 1 | 0 | 0 | 0 | 0 | 5 |
| **S125A-7** | 1 | 2 | 0 | 0 | 0 | 0 | 0 | 3 |
| **Mock-1** | 0 | 0 | 0 | 0 | 0 | 0 | 0 | 0 |
| **Mock-2** | 0 | 0 | 0 | 0 | 0 | 0 | 0 | 0 |
| **Mock-3** | 0 | 0 | 0 | 0 | 0 | 0 | 0 | 0 |
| **Mock-4** | 0 | 0 | 0 | 0 | 0 | 0 | 0 | 0 |
| **Mock-5** | 0 | 0 | 0 | 0 | 0 | 0 | 0 | 0 |
| **Mock-6** | 0 | 0 | 0 | 0 | 0 | 0 | 0 | 0 |
| **Mock-7** | 0 | 0 | 0 | 0 | 0 | 0 | 0 | 0 |

“×” means death.

| Clinical signs record chart | | | | | | | | |  |
| --- | --- | --- | --- | --- | --- | --- | --- | --- | --- |
| 4 day’s post infection | | | | | | | | |  |
|  | | | | | | | | |  |
|  | **ruffled fur**  **（0-5）** | **Hunchbacked** | **slow in action** | **Limb weakness/ Limb trembling** | **Limb paralysis** | **Weight loss** | **Moribund and death** | **sum** | |
|  |  | **（0-5）** | **（0-5）** | **（0-5）** | **（0-5）** | **（0-5）** | **35** | **（0-35）** | |
| **WT-1** | 0 | 0 | 0 | 0 | 0 | 5 | 0 | 5 | |
| **WT-2** | 1 | 1 | 0 | 0 | 0 | 5 | 0 | 7 | |
| **WT-3** | 0 | 0 | 0 | 0 | 0 | 0 | 0 | 0 | |
| **WT-4** | 0 | 0 | 0 | 0 | 0 | 0 | 0 | 0 | |
| **WT-5** | 0 | 0 | 0 | 0 | 0 | 0 | 0 | 0 | |
| **WT-6** | 2 | 3 | 3 | 0 | 0 | 5 | 0 | 13 | |
| **WT-7** | 0 | 0 | 0 | 0 | 0 | 5 | 0 | 5 | |
| **S125A-1** | 2 | 2 | 2 | 1 | 0 | 0 | 0 | 7 | |
| **S125A-2** | 0 | 0 | 0 | 0 | 0 | 0 | 0 | 0 | |
| **S125A-3** | 0 | 0 | 0 | 0 | 0 | 0 | 0 | 0 | |
| **S125A-4** | 0 | 0 | 0 | 0 | 0 | 0 | 0 | 0 | |
| **S125A-5** | 0 | 0 | 0 | 0 | 0 | 0 | 0 | 0 | |
| **S125A-6** | 2 | 3 | 3 | 0 | 0 | 0 | 0 | 8 | |
| **S125A-7** | 2 | 2 | 2 | 0 | 0 | 0 | 0 | 6 | |
| **Mock-1** | 0 | 0 | 0 | 0 | 0 | 0 | 0 | 0 | |
| **Mock-2** | 0 | 0 | 0 | 0 | 0 | 0 | 0 | 0 | |
| **Mock-3** | 0 | 0 | 0 | 0 | 0 | 0 | 0 | 0 | |
| **Mock-4** | 0 | 0 | 0 | 0 | 0 | 5 | 0 | 5 | |
| **Mock-5** | 0 | 0 | 0 | 0 | 0 | 0 | 0 | 0 | |
| **Mock-6** | 0 | 0 | 0 | 0 | 0 | 0 | 0 | 0 | |
| **Mock-7** | 0 | 0 | 0 | 0 | 0 | 0 | 0 | 0 | |

“×” meants death.

| Clinical signs record chart | | | | | | | | |  |
| --- | --- | --- | --- | --- | --- | --- | --- | --- | --- |
| 5 day’s post infection | | | | | | | | |  |
|  | | | | | | | | |  |
|  | **ruffled fur**  **（0-5）** | **Hunchbacked** | **slow in action** | **Limb weakness/ Limb trembling** | **Limb paralysis** | **Weight loss** | **Moribund and death** | **sum** | |
|  |  | **（0-5）** | **（0-5）** | **（0-5）** | **（0-5）** | **（0-5）** | **35** | **（0-35）** | |
| **WT-1** | 1 | 1 | 5 | 5 | 4 | 5 | 0 | 21 | |
| **WT-2** | 0 | 0 | 0 | 0 | 0 | 5 | 0 | 5 | |
| **WT-3** | 0 | 0 | 0 | 0 | 0 | 5 | 0 | 5 | |
| **WT-4** | 0 | 0 | 0 | 0 | 0 | 5 | 0 | 5 | |
| **WT-5** | 0 | 0 | 0 | 0 | 0 | 5 | 0 | 5 | |
| **WT-6** | 3 | 2 | 5 | 5 | 5 | 5 | 0 | 25 | |
| **WT-7** | 3 | 3 | 5 | 5 | 5 | 5 | 0 | 26 | |
| **S125A-1** | 0 | 0 | 0 | 0 | 0 | 0 | 0 | 0 | |
| **S125A-2** | 1 | 2 | 2 | 0 | 0 | 5 | 0 | 10 | |
| **S125A-3** | 0 | 0 | 0 | 0 | 0 | 0 | 0 | 0 | |
| **S125A-4** | 0 | 0 | 0 | 0 | 0 | 5 | 0 | 5 | |
| **S125A-5** | 0 | 0 | 0 | 0 | 0 | 0 | 0 | 0 | |
| **S125A-6** | 2 | 2 | 1 | 1 | 0 | 0 | 0 | 6 | |
| **S125A-7** | 0 | 0 | 0 | 0 | 0 | 0 | 0 | 0 | |
| **Mock-1** | 0 | 0 | 0 | 0 | 0 | 0 | 0 | 0 | |
| **Mock-2** | 0 | 0 | 0 | 0 | 0 | 5 | 0 | 5 | |
| **Mock-3** | 0 | 0 | 0 | 0 | 0 | 0 | 0 | 0 | |
| **Mock-4** | 0 | 0 | 0 | 0 | 0 | 0 | 0 | 0 | |
| **Mock-5** | 0 | 0 | 0 | 0 | 0 | 0 | 0 | 0 | |
| **Mock-6** | 0 | 0 | 0 | 0 | 0 | 0 | 0 | 0 | |
| **Mock-7** | 0 | 0 | 0 | 0 | 0 | 0 | 0 | 0 | |

“×” means death.

| Clinical signs record chart | | | | | | | | |  |
| --- | --- | --- | --- | --- | --- | --- | --- | --- | --- |
| 6 day’s post infection | | | | | | | | |  |
|  | | | | | | | | |  |
|  | **ruffled fur**  **（0-5）** | **Hunchbacked** | **slow in action** | **Limb weakness/ Limb trembling** | **Limb paralysis** | **Weight loss** | **Moribund and death** | **sum** | |
|  |  | **（0-5）** | **（0-5）** | **（0-5）** | **（0-5）** | **（0-5）** | **35** | **（0-35）** | |
| **WT-1** | 3 | 0 | 5 | 5 | 5 | 5 | 0 | 23 | |
| **WT-2** | 0 | 0 | 0 | 0 | 0 | 0 | 0 | 0 | |
| **WT-3** | 1 | 2 | 3 | 2 | 0 | 5 | 0 | 13 | |
| **WT-4** | 0 | 0 | 0 | 0 | 0 | 0 | 0 | 0 | |
| **WT-5** | 0 | 0 | 0 | 0 | 0 | 0 | 0 | 0 | |
| **WT-6** | 3 | 2 | 5 | 4 | 4 | 5 | 0 | 23 | |
| **WT-7** | 2 | 3 | 5 | 5 | 5 | 5 | 0 | 25 | |
| **S125A-1** | 0 | 0 | 0 | 0 | 0 | 0 | 0 | 0 | |
| **S125A-2** | 1 | 3 | 1 | 0 | 0 | 0 | 0 | 5 | |
| **S125A-3** | 0 | 0 | 0 | 0 | 0 | 0 | 0 | 0 | |
| **S125A-4** | 0 | 0 | 0 | 0 | 0 | 0 | 0 | 0 | |
| **S125A-5** | 0 | 0 | 0 | 0 | 0 | 0 | 0 | 0 | |
| **S125A-6** | 1 | 2 | 1 | 0 | 0 | 0 | 0 | 4 | |
| **S125A-7** | 0 | 0 | 0 | 0 | 0 | 0 | 0 | 0 | |
| **Mock-1** | 0 | 0 | 0 | 0 | 0 | 0 | 0 | 0 | |
| **Mock-2** | 0 | 0 | 0 | 0 | 0 | 0 | 0 | 0 | |
| **Mock-3** | 0 | 0 | 0 | 0 | 0 | 0 | 0 | 0 | |
| **Mock-4** | 0 | 0 | 0 | 0 | 0 | 0 | 0 | 0 | |
| **Mock-5** | 0 | 0 | 0 | 0 | 0 | 0 | 0 | 0 | |
| **Mock-6** | 0 | 0 | 0 | 0 | 0 | 0 | 0 | 0 | |
| **Mock-7** | 0 | 0 | 0 | 0 | 0 | 0 | 0 | 0 | |

“×” means death.

| Clinical signs record chart | | | | | | | | |  |
| --- | --- | --- | --- | --- | --- | --- | --- | --- | --- |
| 7 day’s post infection | | | | | | | | |  |
|  | | | | | | | | |  |
|  | **ruffled fur**  **（0-5）** | **Hunchbacked** | **slow in action** | **Limb weakness/ Limb trembling** | **Limb paralysis** | **Weight loss** | **Moribund and death** | **sum** | |
|  |  | **（0-5）** | **（0-5）** | **（0-5）** | **（0-5）** | **（0-5）** | **35** | **（0-35）** | |
| **WT-1** | 0 | 0 | 0 | 0 | 0 | 0 | 35 | 35 | |
| **WT-2** | 0 | 0 | 0 | 0 | 0 | 5 | 0 | 5 | |
| **WT-3** | 2 | 2 | 4 | 3 | 3 | 5 | 0 | 19 | |
| **WT-4** | 0 | 0 | 0 | 0 | 0 | 5 | 0 | 5 | |
| **WT-5** | 0 | 0 | 0 | 0 | 0 | 5 | 0 | 5 | |
| **WT-6** | 0 | 0 | 0 | 0 | 0 | 0 | 35 | 35 | |
| **WT-7** | 0 | 0 | 0 | 0 | 0 | 0 | 35 | 35 | |
| **S125A-1** | 0 | 0 | 0 | 0 | 0 | 0 | 0 | 0 | |
| **S125A-2** | 0 | 0 | 0 | 0 | 0 | 0 | 0 | 0 | |
| **S125A-3** | 0 | 0 | 0 | 0 | 0 | 0 | 5 | 5 | |
| **S125A-4** | 0 | 0 | 0 | 0 | 0 | 0 | 0 | 0 | |
| **S125A-5** | 0 | 0 | 0 | 0 | 0 | 0 | 0 | 0 | |
| **S125A-6** | 0 | 0 | 0 | 0 | 0 | 0 | 0 | 0 | |
| **S125A-7** | 0 | 0 | 0 | 0 | 0 | 0 | 0 | 0 | |
| **Mock-1** | 0 | 0 | 0 | 0 | 0 | 5 | 0 | 5 | |
| **Mock-2** | 0 | 0 | 0 | 0 | 0 | 0 | 0 | 0 | |
| **Mock-3** | 0 | 0 | 0 | 0 | 0 | 0 | 0 | 0 | |
| **Mock-4** | 0 | 0 | 0 | 0 | 0 | 0 | 0 | 0 | |
| **Mock-5** | 0 | 0 | 0 | 0 | 0 | 0 | 0 | 0 | |
| **Mock-6** | 0 | 0 | 0 | 0 | 0 | 0 | 0 | 0 | |
| **Mock-7** | 0 | 0 | 0 | 0 | 0 | 0 | 0 | 0 | |

“×” means death.

| Clinical signs record chart | | | | | | | | |  |
| --- | --- | --- | --- | --- | --- | --- | --- | --- | --- |
| 8 day’s post infection | | | | | | | | |  |
|  | | | | | | | | |  |
|  | **ruffled fur**  **（0-5）** | **Hunchbacked** | **slow in action** | **Limb weakness/ Limb trembling** | **Limb paralysis** | **Weight loss** | **Moribund and death** | **sum** | |
|  |  | **（0-5）** | **（0-5）** | **（0-5）** | **（0-5）** | **（0-5）** | **35** | **（0-35）** | |
| **WT-1** | × | × | × | × | × | × | × | × | |
| **WT-2** | 0 | 0 | 0 | 0 | 0 | 0 | 0 | 0 | |
| **WT-3** | 3 | 2 | 4 | 4 | 4 | 5 | 0 | 22 | |
| **WT-4** | 0 | 0 | 0 | 0 | 0 | 0 | 0 | 0 | |
| **WT-5** | 0 | 0 | 0 | 0 | 0 | 0 | 0 | 0 | |
| **WT-6** | × | × | × | × | × | × | × | × | |
| **WT-7** | × | × | × | × | × | × | × | × | |
| **S125A-1** | 0 | 0 | 0 | 0 | 0 | 0 | 0 | 0 | |
| **S125A-2** | 0 | 0 | 0 | 0 | 0 | 0 | 0 | 0 | |
| **S125A-3** | 0 | 0 | 0 | 0 | 0 | 0 | 0 | 0 | |
| **S125A-4** | 0 | 0 | 0 | 0 | 0 | 0 | 0 | 0 | |
| **S125A-5** | 0 | 0 | 0 | 0 | 0 | 0 | 0 | 0 | |
| **S125A-6** | 0 | 0 | 0 | 0 | 0 | 0 | 0 | 0 | |
| **S125A-7** | 0 | 0 | 0 | 0 | 0 | 0 | 0 | 0 | |
| **Mock-1** | 0 | 0 | 0 | 0 | 0 | 0 | 0 | 0 | |
| **Mock-2** | 0 | 0 | 0 | 0 | 0 | 5 | 0 | 5 | |
| **Mock-3** | 0 | 0 | 0 | 0 | 0 | 0 | 0 | 0 | |
| **Mock-4** | 0 | 0 | 0 | 0 | 0 | 0 | 0 | 0 | |
| **Mock-5** | 0 | 0 | 0 | 0 | 0 | 0 | 0 | 0 | |
| **Mock-6** | 0 | 0 | 0 | 0 | 0 | 0 | 0 | 0 | |
| **Mock-7** | 0 | 0 | 0 | 0 | 0 | 0 | 0 | 0 | |

“×” means death.

| Clinical signs record chart | | | | | | | | |  |
| --- | --- | --- | --- | --- | --- | --- | --- | --- | --- |
| 9 day’s post infection | | | | | | | | |  |
|  | | | | | | | | |  |
|  | **ruffled fur**  **（0-5）** | **Hunchbacked** | **slow in action** | **Limb weakness/ Limb trembling** | **Limb paralysis** | **Weight loss** | **Moribund and death** | **sum** | |
|  |  | **（0-5）** | **（0-5）** | **（0-5）** | **（0-5）** | **（0-5）** | **35** | **（0-35）** | |
| **WT-1** | × | × | × | × | × | × | × | × | |
| **WT-2** | 0 | 0 | 0 | 0 | 0 | 0 | 0 | 0 | |
| **WT-3** | 2 | 1 | 3 | 3 | 4 | 5 | 0 | 18 | |
| **WT-4** | 0 | 0 | 0 | 0 | 0 | 0 | 0 | 0 | |
| **WT-5** | 0 | 0 | 0 | 0 | 0 | 5 | 0 | 5 | |
| **WT-6** | × | × | × | × | × | × | × | × | |
| **WT-7** | × | × | × | × | × | × | × | × | |
| **S125A-1** | 0 | 0 | 0 | 0 | 0 | 0 | 0 | 0 | |
| **S125A-2** | 0 | 0 | 0 | 0 | 0 | 0 | 0 | 0 | |
| **S125A-3** | 0 | 0 | 0 | 0 | 0 | 0 | 0 | 0 | |
| **S125A-4** | 0 | 0 | 0 | 0 | 0 | 0 | 0 | 0 | |
| **S125A-5** | 0 | 0 | 0 | 0 | 0 | 0 | 0 | 0 | |
| **S125A-6** | 0 | 0 | 0 | 0 | 0 | 0 | 0 | 0 | |
| **S125A-7** | 0 | 0 | 0 | 0 | 0 | 0 | 0 | 0 | |
| **Mock-1** | 0 | 0 | 0 | 0 | 0 | 0 | 0 | 0 | |
| **Mock-2** | 0 | 0 | 0 | 0 | 0 | 0 | 0 | 0 | |
| **Mock-3** | 0 | 0 | 0 | 0 | 0 | 0 | 0 | 0 | |
| **Mock-4** | 0 | 0 | 0 | 0 | 0 | 0 | 0 | 0 | |
| **Mock-5** | 0 | 0 | 0 | 0 | 0 | 0 | 0 | 0 | |
| **Mock-6** | 0 | 0 | 0 | 0 | 0 | 0 | 0 | 0 | |
| **Mock-7** | 0 | 0 | 0 | 0 | 0 | 0 | 0 | 0 | |

“×” means death.

| Clinical signs record chart | | | | | | | | |  |
| --- | --- | --- | --- | --- | --- | --- | --- | --- | --- |
| 10 day’s post infection | | | | | | | | |  |
|  | | | | | | | | |  |
|  | **ruffled fur**  **（0-5）** | **Hunchbacked** | **slow in action** | **Limb weakness/ Limb trembling** | **Limb paralysis** | **Weight loss** | **Moribund and death** | **sum** | |
|  |  | **（0-5）** | **（0-5）** | **（0-5）** | **（0-5）** | **（0-5）** | **35** | **（0-35）** | |
| **WT-1** | × | × | × | × | × | × | × | × | |
| **WT-2** | 1 | 1 | 2 | 3 | 4 | 0 | 0 | 11 | |
| **WT-3** | 0 | 0 | 0 | 0 | 0 | 0 | 0 | 0 | |
| **WT-4** | 0 | 0 | 0 | 0 | 0 | 0 | 0 | 0 | |
| **WT-5** | 0 | 0 | 0 | 0 | 0 | 0 | 0 | 0 | |
| **WT-6** | × | × | × | × | × | × | × | × | |
| **WT-7** | × | × | × | × | × | × | × | × | |
| **S125A-1** | 0 | 0 | 0 | 0 | 0 | 0 | 0 | 0 | |
| **S125A-2** | 0 | 0 | 0 | 0 | 0 | 0 | 0 | 0 | |
| **S125A-3** | 0 | 0 | 0 | 0 | 0 | 0 | 0 | 0 | |
| **S125A-4** | 0 | 0 | 0 | 0 | 0 | 0 | 0 | 0 | |
| **S125A-5** | 0 | 0 | 0 | 0 | 0 | 0 | 0 | 0 | |
| **S125A-6** | 0 | 0 | 0 | 0 | 0 | 5 | 0 | 5 | |
| **S125A-7** | 0 | 0 | 0 | 0 | 0 | 0 | 0 | 0 | |
| **Mock-1** | 0 | 0 | 0 | 0 | 0 | 5 | 0 | 5 | |
| **Mock-2** | 0 | 0 | 0 | 0 | 0 | 0 | 0 | 0 | |
| **Mock-3** | 0 | 0 | 0 | 0 | 0 | 0 | 0 | 0 | |
| **Mock-4** | 0 | 0 | 0 | 0 | 0 | 5 | 0 | 5 | |
| **Mock-5** | 0 | 0 | 0 | 0 | 0 | 0 | 0 | 0 | |
| **Mock-6** | 0 | 0 | 0 | 0 | 0 | 0 | 0 | 0 | |
| **Mock-7** | 0 | 0 | 0 | 0 | 0 | 0 | 0 | 0 | |

“×” means death.

| Clinical signs record chart | | | | | | | | |  |
| --- | --- | --- | --- | --- | --- | --- | --- | --- | --- |
| 11 day’s post infection | | | | | | | | |  |
|  | | | | | | | | |  |
|  | **ruffled fur**  **（0-5）** | **Hunchbacked** | **slow in action** | **Limb weakness/ Limb trembling** | **Limb paralysis** | **Weight loss** | **Moribund and death** | **sum** | |
|  |  | **（0-5）** | **（0-5）** | **（0-5）** | **（0-5）** | **（0-5）** | **35** | **（0-35）** | |
| **WT-1** | × | × | × | × | × | × | × | × | |
| **WT-2** | 0 | 0 | 0 | 0 | 0 | 5 | 0 | 5 | |
| **WT-3** | 1 | 2 | 2 | 2 | 2 | 0 | 0 | 9 | |
| **WT-4** | 0 | 0 | 0 | 0 | 0 | 5 | 0 | 5 | |
| **WT-5** | 0 | 0 | 0 | 0 | 0 | 0 | 0 | 0 | |
| **WT-6** | × | × | × | × | × | × | × | × | |
| **WT-7** | × | × | × | × | × | × | × | × | |
| **S125A-1** | 0 | 0 | 0 | 0 | 0 | 5 | 0 | 5 | |
| **S125A-2** | 0 | 0 | 0 | 0 | 0 | 5 | 0 | 5 | |
| **S125A-3** | 0 | 0 | 0 | 0 | 0 | 0 | 0 | 0 | |
| **S125A-4** | 0 | 0 | 0 | 0 | 0 | 5 | 0 | 5 | |
| **S125A-5** | 0 | 0 | 0 | 0 | 0 | 0 | 0 | 0 | |
| **S125A-6** | 0 | 0 | 0 | 0 | 0 | 5 | 0 | 5 | |
| **S125A-7** | 0 | 0 | 0 | 0 | 0 | 0 | 0 | 0 | |
| **Mock-1** | 0 | 0 | 0 | 0 | 0 | 5 | 0 | 5 | |
| **Mock-2** | 0 | 0 | 0 | 0 | 0 | 0 | 0 | 0 | |
| **Mock-3** | 0 | 0 | 0 | 0 | 0 | 0 | 0 | 0 | |
| **Mock-4** | 0 | 0 | 0 | 0 | 0 | 0 | 0 | 0 | |
| **Mock-5** | 0 | 0 | 0 | 0 | 0 | 5 | 0 | 5 | |
| **Mock-6** | 0 | 0 | 0 | 0 | 0 | 0 | 0 | 0 | |
| **Mock-7** | 0 | 0 | 0 | 0 | 0 | 0 | 0 | 0 | |

“×” means death.

| Clinical signs record chart | | | | | | | | |  |
| --- | --- | --- | --- | --- | --- | --- | --- | --- | --- |
| 12 day’s post infection | | | | | | | | |  |
|  | | | | | | | | |  |
|  | **ruffled fur**  **（0-5）** | **Hunchbacked** | **slow in action** | **Limb weakness/ Limb trembling** | **Limb paralysis** | **Weight loss** | **Moribund and death** | **sum** | |
|  |  | **（0-5）** | **（0-5）** | **（0-5）** | **（0-5）** | **（0-5）** | **35** | **（0-35）** | |
| **WT-1** | × | × | × | × | × | × | × | × | |
| **WT-2** | 0 | 0 | 0 | 0 | 0 | 0 | 0 | 0 | |
| **WT-3** | 1 | 1 | 1 | 2 | 2 | 0 | 0 | 7 | |
| **WT-4** | 0 | 0 | 0 | 0 | 0 | 0 | 0 | 0 | |
| **WT-5** | 0 | 0 | 0 | 0 | 0 | 5 | 0 | 5 | |
| **WT-6** | × | × | × | × | × | × | × | × | |
| **WT-7** | × | × | × | × | × | × | × | × | |
| **S125A-1** | 0 | 0 | 0 | 0 | 0 | 0 | 0 | 0 | |
| **S125A-2** | 0 | 0 | 0 | 0 | 0 | 0 | 0 | 0 | |
| **S125A-3** | 0 | 0 | 0 | 0 | 0 | 0 | 0 | 0 | |
| **S125A-4** | 0 | 0 | 0 | 0 | 0 | 0 | 0 | 0 | |
| **S125A-5** | 0 | 0 | 0 | 0 | 0 | 5 | 0 | 5 | |
| **S125A-6** | 0 | 0 | 0 | 0 | 0 | 5 | 0 | 5 | |
| **S125A-7** | 0 | 0 | 0 | 0 | 0 | 0 | 0 | 0 | |
| **Mock-1** | 0 | 0 | 0 | 0 | 0 | 5 | 0 | 5 | |
| **Mock-2** | 0 | 0 | 0 | 0 | 0 | 0 | 0 | 0 | |
| **Mock-3** | 0 | 0 | 0 | 0 | 0 | 0 | 0 | 0 | |
| **Mock-4** | 0 | 0 | 0 | 0 | 0 | 0 | 0 | 0 | |
| **Mock-5** | 0 | 0 | 0 | 0 | 0 | 0 | 0 | 0 | |
| **Mock-6** | 0 | 0 | 0 | 0 | 0 | 0 | 0 | 0 | |
| **Mock-7** | 0 | 0 | 0 | 0 | 0 | 0 | 0 | 0 | |

“×” means death.

| Clinical signs record chart | | | | | | | | |  |
| --- | --- | --- | --- | --- | --- | --- | --- | --- | --- |
| 13 day’s post infection | | | | | | | | |  |
|  | | | | | | | | |  |
|  | **ruffled fur**  **（0-5）** | **Hunchbacked** | **slow in action** | **Limb weakness/ Limb trembling** | **Limb paralysis** | **Weight loss** | **Moribund and death** | **sum** | |
|  |  | **（0-5）** | **（0-5）** | **（0-5）** | **（0-5）** | **（0-5）** | **35** | **（0-35）** | |
| **WT-1** | × | × | × | × | × | × | × | × | |
| **WT-2** | 0 | 0 | 0 | 0 | 0 | 0 | 0 | 0 | |
| **WT-3** | 1 | 0 | 1 | 2 | 2 | 0 | 0 | 6 | |
| **WT-4** | 0 | 0 | 0 | 0 | 0 | 0 | 0 | 0 | |
| **WT-5** | 0 | 0 | 0 | 0 | 0 | 0 | 0 | 0 | |
| **WT-6** | × | × | × | × | × | × | × | × | |
| **WT-7** | × | × | × | × | × | × | × | × | |
| **S125A-1** | 0 | 0 | 0 | 0 | 0 | 0 | 0 | 0 | |
| **S125A-2** | 0 | 0 | 0 | 0 | 0 | 0 | 0 | 0 | |
| **S125A-3** | 0 | 0 | 0 | 0 | 0 | 0 | 0 | 0 | |
| **S125A-4** | 0 | 0 | 0 | 0 | 0 | 0 | 0 | 0 | |
| **S125A-5** | 0 | 0 | 0 | 0 | 0 | 0 | 0 | 0 | |
| **S125A-6** | 0 | 0 | 0 | 0 | 0 | 0 | 0 | 0 | |
| **S125A-7** | 0 | 0 | 0 | 0 | 0 | 0 | 0 | 0 | |
| **Mock-1** | 0 | 0 | 0 | 0 | 0 | 0 | 0 | 0 | |
| **Mock-2** | 0 | 0 | 0 | 0 | 0 | 0 | 0 | 0 | |
| **Mock-3** | 0 | 0 | 0 | 0 | 0 | 0 | 0 | 0 | |
| **Mock-4** | 0 | 0 | 0 | 0 | 0 | 0 | 0 | 0 | |
| **Mock-5** | 0 | 0 | 0 | 0 | 0 | 0 | 0 | 0 | |
| **Mock-6** | 0 | 0 | 0 | 0 | 0 | 0 | 0 | 0 | |
| **Mock-7** | 0 | 0 | 0 | 0 | 0 | 0 | 0 | 0 | |

“×” means death.

| Clinical signs record chart | | | | | | | | |  |
| --- | --- | --- | --- | --- | --- | --- | --- | --- | --- |
| 14 day’s post infection | | | | | | | | |  |
|  | | | | | | | | |  |
|  | **ruffled fur**  **（0-5）** | **Hunchbacked** | **slow in action** | **Limb weakness/ Limb trembling** | **Limb paralysis** | **Weight loss** | **Moribund and death** | **sum** | |
|  |  | **（0-5）** | **（0-5）** | **（0-5）** | **（0-5）** | **（0-5）** | **35** | **（0-35）** | |
| **WT-1** | × | × | × | × | × | × | × | × | |
| **WT-2** | 0 | 0 | 0 | 0 | 0 | 0 | 0 | 0 | |
| **WT-3** | 0 | 0 | 1 | 2 | 1 | 0 | 0 | 4 | |
| **WT-4** | 0 | 0 | 0 | 0 | 0 | 0 | 0 | 0 | |
| **WT-5** | 0 | 0 | 0 | 0 | 0 | 0 | 0 | 0 | |
| **WT-6** | × | × | × | × | × | × | × | × | |
| **WT-7** | × | × | × | × | × | × | × | × | |
| **S125A-1** | 0 | 0 | 0 | 0 | 0 | 0 | 0 | 0 | |
| **S125A-2** | 0 | 0 | 0 | 0 | 0 | 0 | 0 | 0 | |
| **S125A-3** | 0 | 0 | 0 | 0 | 0 | 0 | 0 | 0 | |
| **S125A-4** | 0 | 0 | 0 | 0 | 0 | 0 | 0 | 0 | |
| **S125A-5** | 0 | 0 | 0 | 0 | 0 | 0 | 0 | 0 | |
| **S125A-6** | 0 | 0 | 0 | 0 | 0 | 5 | 0 | 5 | |
| **S125A-7** | 0 | 0 | 0 | 0 | 0 | 0 | 0 | 0 | |
| **Mock-1** | 0 | 0 | 0 | 0 | 0 | 0 | 0 | 0 | |
| **Mock-2** | 0 | 0 | 0 | 0 | 0 | 0 | 0 | 0 | |
| **Mock-3** | 0 | 0 | 0 | 0 | 0 | 0 | 0 | 0 | |
| **Mock-4** | 0 | 0 | 0 | 0 | 0 | 0 | 0 | 0 | |
| **Mock-5** | 0 | 0 | 0 | 0 | 0 | 0 | 0 | 0 | |
| **Mock-6** | 0 | 0 | 0 | 0 | 0 | 0 | 0 | 0 | |
| **Mock-7** | 0 | 0 | 0 | 0 | 0 | 0 | 0 | 0 | |

“×” means death.

# Supplementary Figure 1


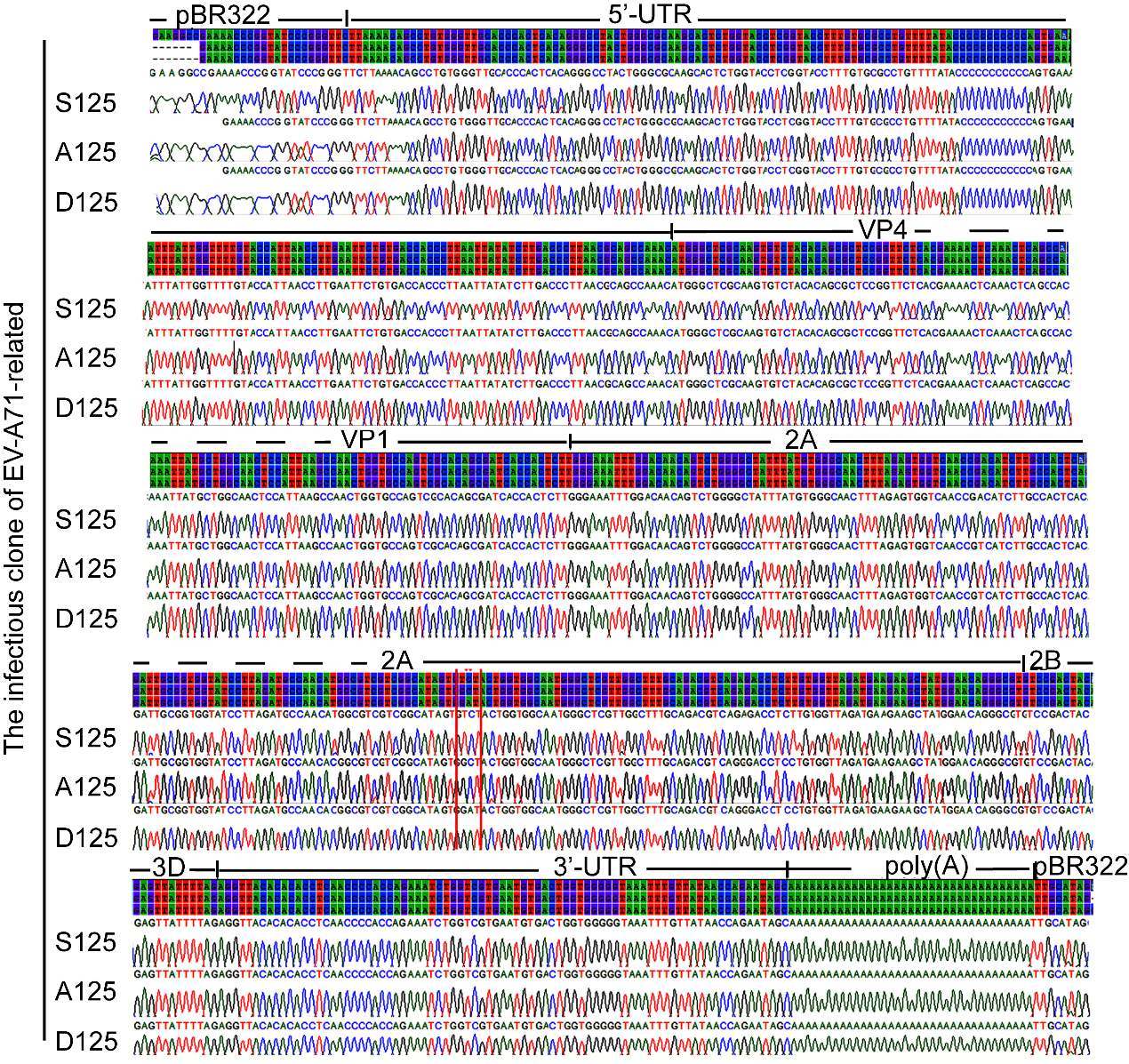
 **Supplementary Figure 1** The Sanger sequencing of EV-A71-related infectious clones. The sequence of EV-A71-related infectious clones pBR322 EV-A71 S125, pBR322 EV-A71 A125, pBR322 EV-A71 D125 were sequenced using Sanger sequencing.

# Supplementary Figure 2


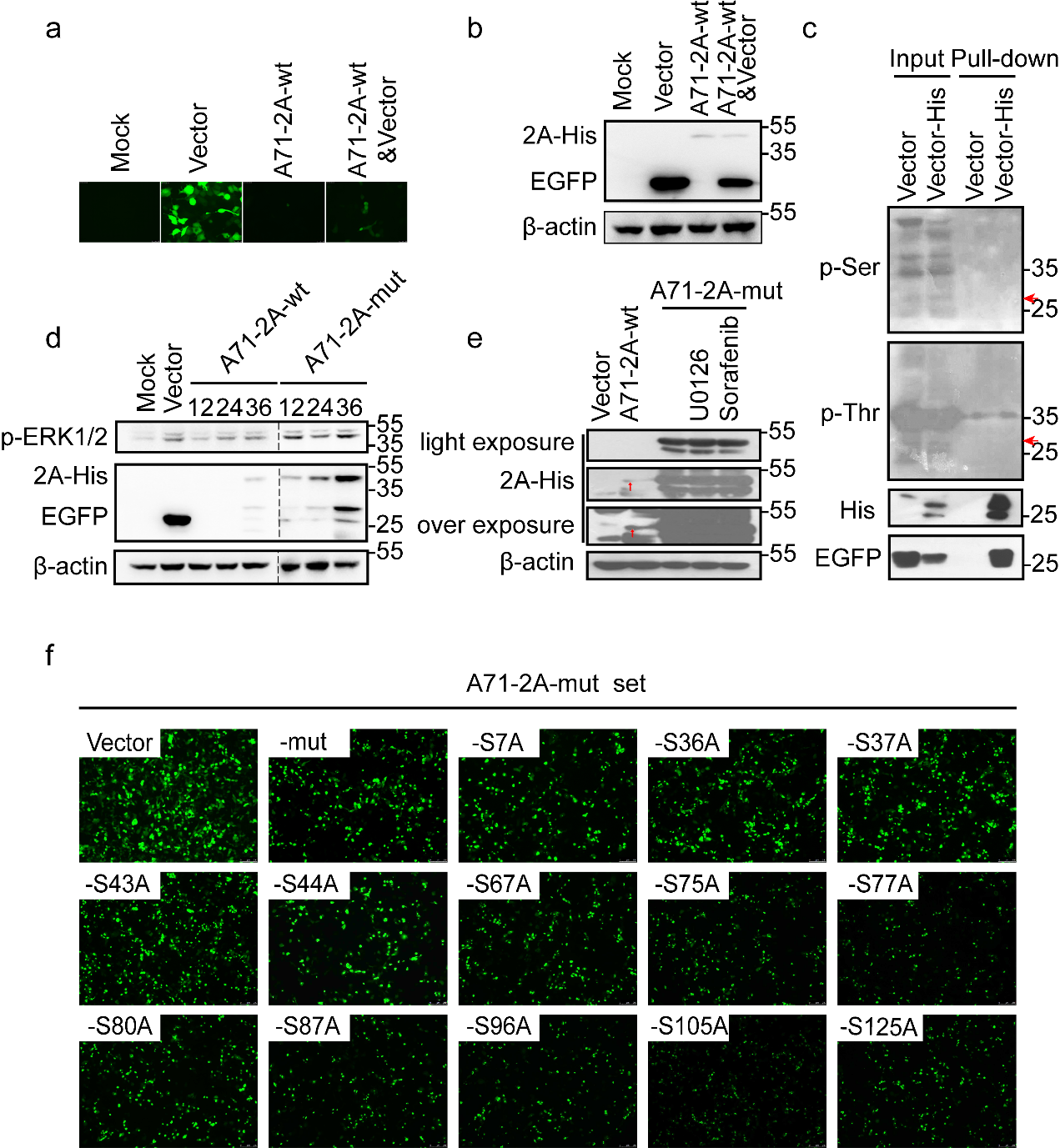
 **Supplementary Figure 2 a,b,** The less EGFP-A71-2A-wt fusion protein expression due to eIF4G-cleavage-mediaed cap-dependent translation shutdown examined by fluorescence microscope. Cells transfected with A71-2A-wt, A71-2A-wt & Vector were examined by fluorescence microscope(a) firstly, then were collected for WB with anti-EGFP and β-actin(b). **c,** No phosphorylation was observed at EGFP-tag. Cell lysates pre-transfected with the vector were applied for his-pulldown and subjected to immunoblotting with anti-EGFP, anti-His, -p-Ser, and -p-Thr antibodies. **d,** ERKs maintained activation, post-transfectied with A71-2A-wt and A71-2A-mut tested by WB. **e,** The U0126 (30 μM) and Sorafenib (2 μM) had no effects on the expression of A71-2A-mut. f, S-to-A mutation had no effect on the expression of A71-2A-mut set.

# Supplementary Figure 3


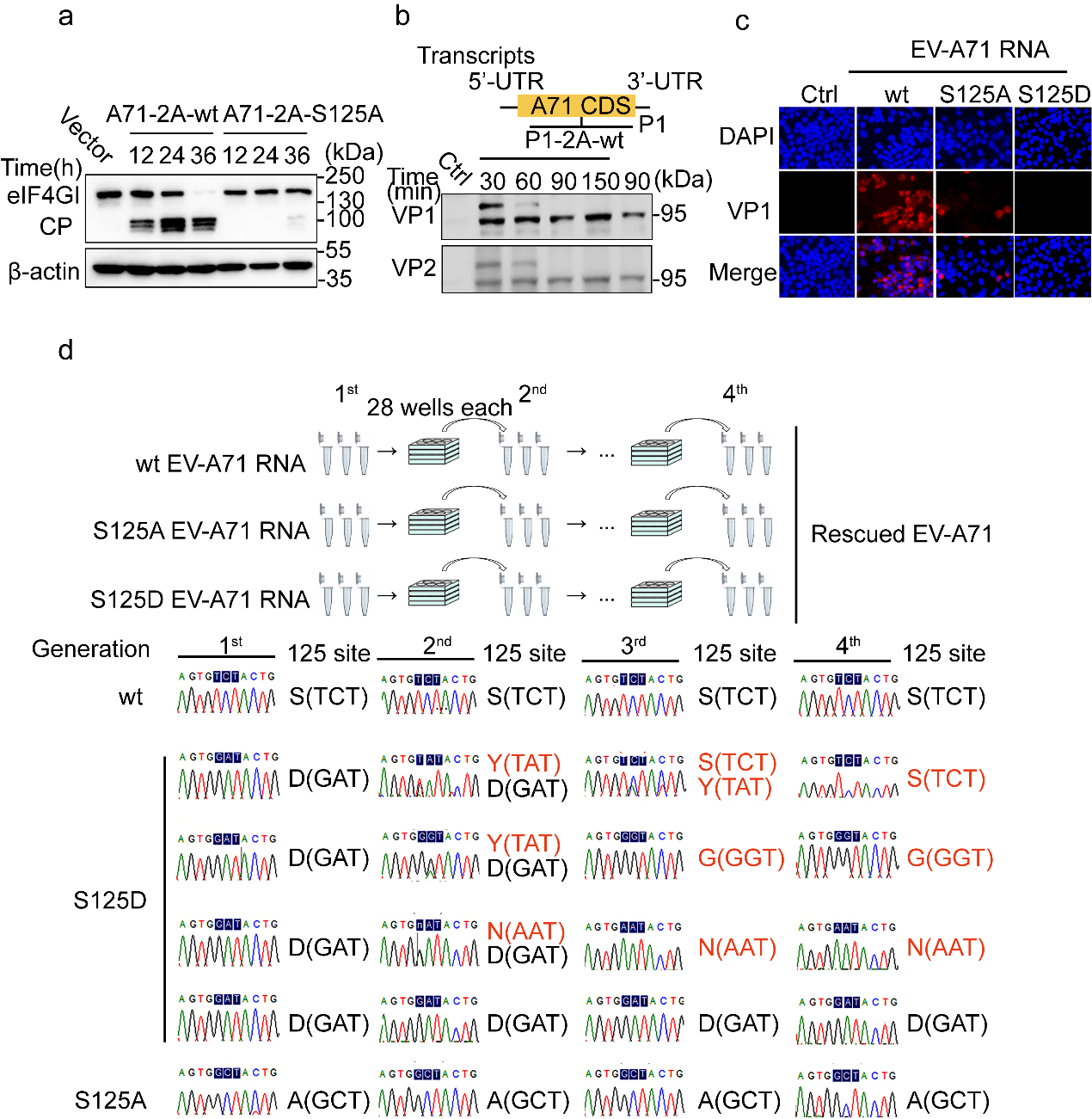


**Supplementary Figure 3** **a,** A71-2A-wt and A71-2A-S125A toward eIF4GI trans-cleavage post-transfection at 12, 24, or 36 h. HEK 293 cells transfected with 2 μg of A71-2A-wt or A71-2A-S125A or Vector as control were harvested and lysed at 12, 24 and 36 h post transfection respectively for WB analysis. **b,** P1-2A cis-cleavage in eukaryotic system. Cis-cleavage activity by P1-2A protein was observed from RNAs in vitro transcribed from pIRES P1-2A-wt in eukaryotic system. **c,** The VP1 expression observed by fluorescence microscope when cells transfected with wt, S125A and S125D EV-A71 genomic RNAs. RD cells transfected with EV-A71 wt, S125A and S125D genomic RNAs for 24 h were fixed in 4% paraformaldehyde, permeabilized with 0.1% triton-x100, and stained with anti-VP1 (red) and DAPI-stained nuclei (blue) and then were observed by confocal microscopy. (Magnification 200X). **d,** The rescued progeny viruses from wt, S125A and S125D EV-A71 genomic RNAs were partly used for the serial passages, and partly for the Sanger sequencing.

# Supplementary Figure 4


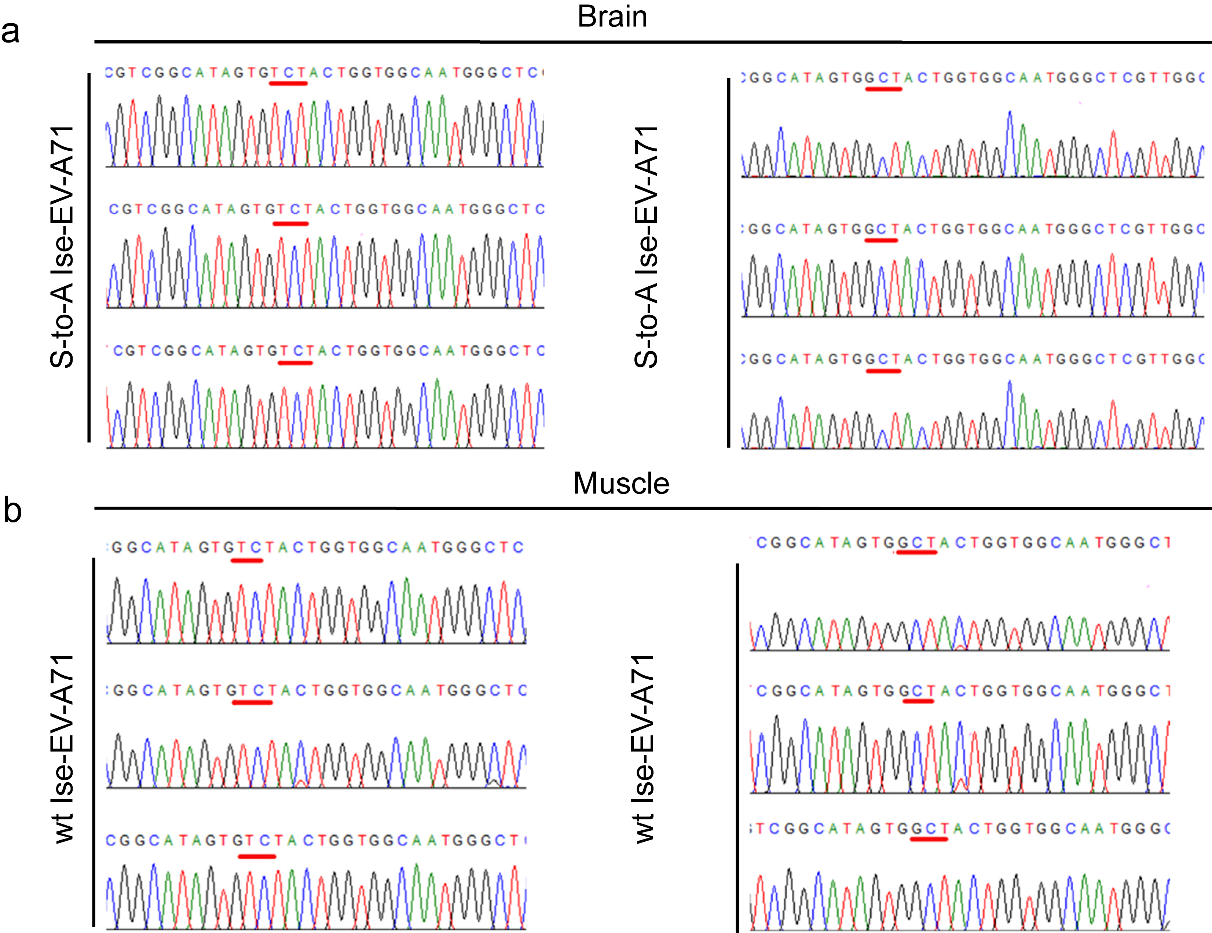


**Supplementary Figure 4** The sequences of wt and S-to-A Ise-EV-A71 from the brains and muscles of mice by Sanger sequencing.

# Supplementary Figure 5


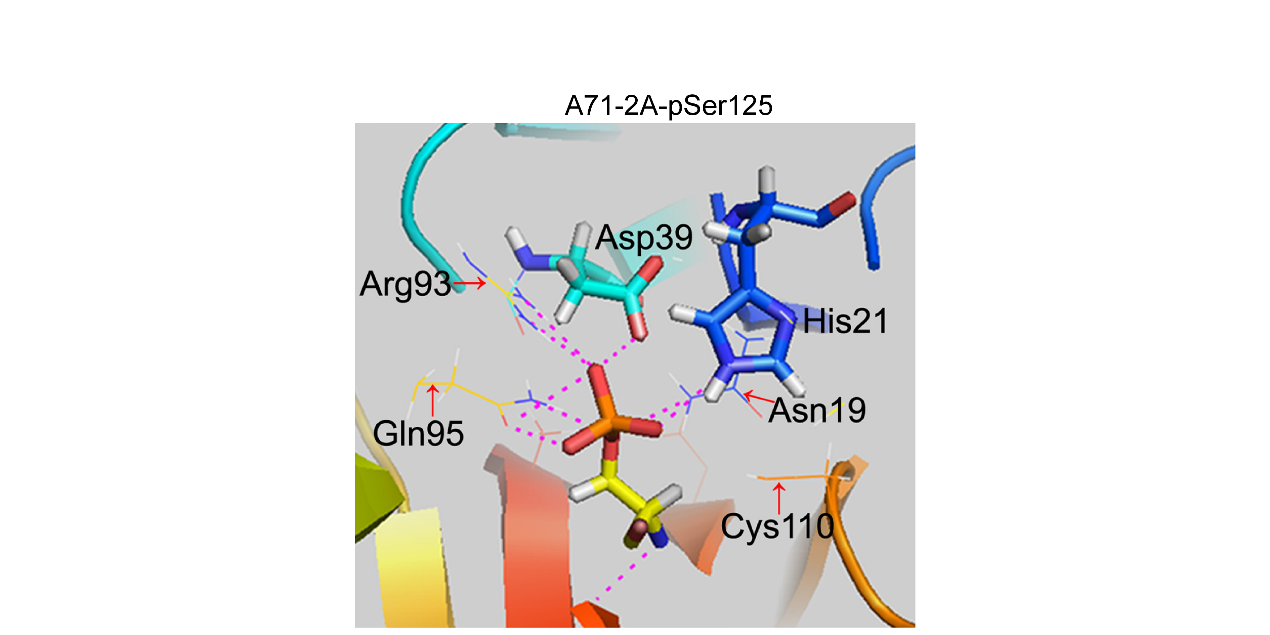


**Supplementary Figure 5** The superimposed structures of simulated EV-A71 2A with Ser125 phosphorylation (A71-2A-pSer125). pSer125 and His21, Asp39 and Cys110 shown in sticks and Arg93, Gln95 and Asn19 shown in lines. The HB formed by pSer125 and Arg93, Gln95 and Asn19 with hydrogen bonds is shown in dashed magenta lines.

# Supplementary Figure 6


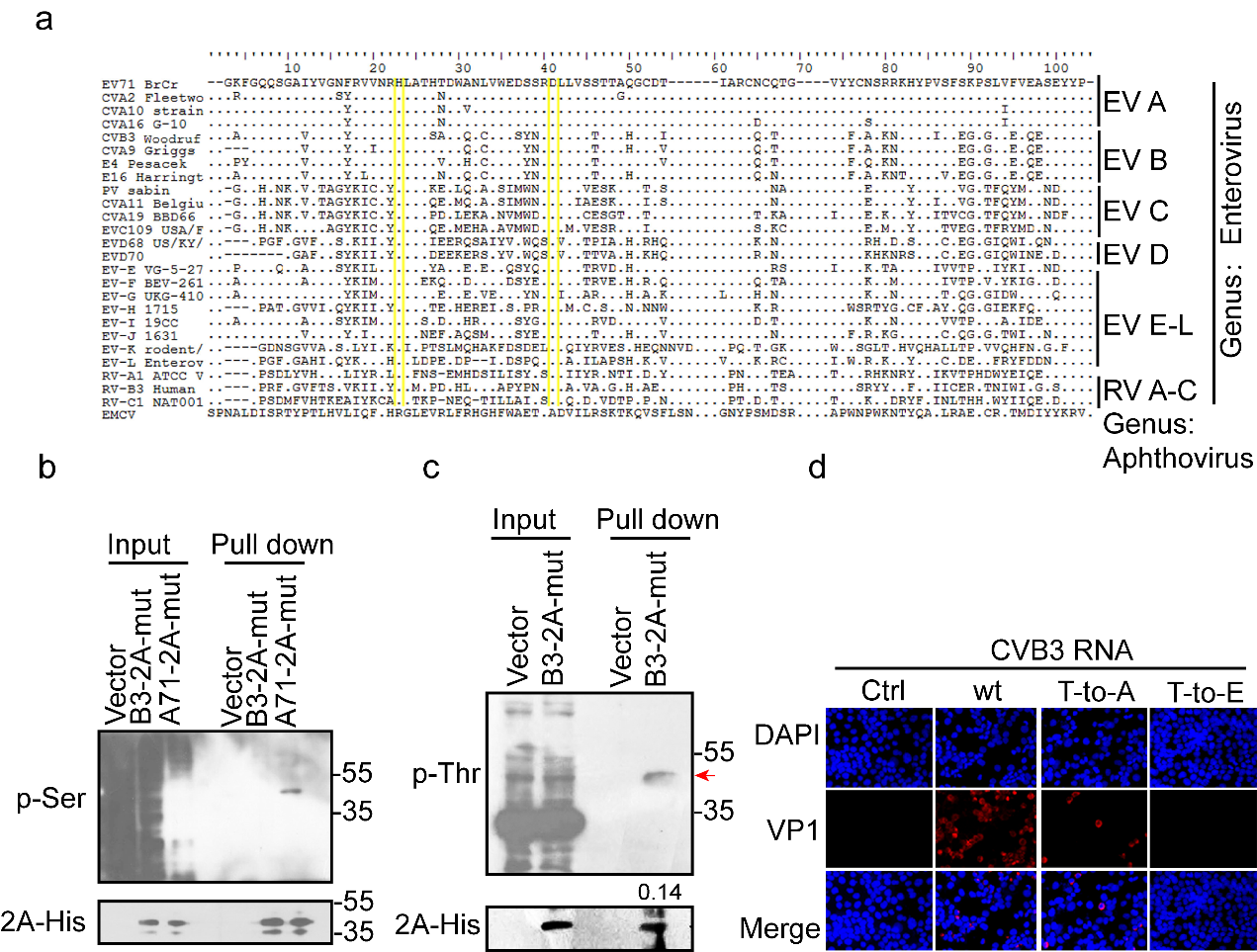


**Supplementary Figure 6a,** Ser/Thr125 was conserved in the *Enterovirus* ranging from EV A-L to RV A-C by MEGA (version 5.05). The relevant sequence from *Aphthovirus* (family *Picornaviridae*) as a control in parallel . The His-Asp-Cys catalytic triad in yellow. **b,c,** The A71-2A-mut and B3-2A-mut phosphorylation by his-pull down and subjected to WB with anti-His and p-Ser and p-Thr antibodies. **d,** Fluorescence intensity of VP1(red) were examined by fluorescence microscopy (200×) in cells transfected with wt, T-to-A and T-to-E genomic RNAs of CVB3.
